# *DDX3Y* gene rescue of a Y chromosome *AZFa* deletion restores germ cell formation and transcriptional programs

**DOI:** 10.1038/srep15041

**Published:** 2015-10-12

**Authors:** Cyril Ramathal, Benjamin Angulo, Meena Sukhwani, Jun Cui, Jens Durruthy-Durruthy, Fang Fang, Paula Schanes, Paul J. Turek, Kyle E. Orwig, Renee Reijo Pera

**Affiliations:** 1 Institute for Stem Cell Biology and Regenerative Medicine & Department of Genetics, Stanford University, Stanford, CA, USA; 2 Departments of Cell Biology & Neuroscience and Chemistry and Biochemistry, Montana State University, Bozeman, MT, USA; 3 The Turek Clinic, San Francisco, CA 94133, USA; 4 Magee Women’s Research Institute, University of Pittsburgh, Pittsburgh, PA, 15260, USA

## Abstract

Deletions of the *AZFa* region (*AZoospermia Factor-a*) region of the human Y chromosome cause irreversible spermatogenic failure that presents clinically in men as Sertoli-cell only (SCO) pathology of the testis. Deletions of the *AZFa* region typically encompass two genes: *DDX3Y* and *USP9Y*. However, human genetic evidence indicates that SCO is most tightly linked to deletion of *DDX3Y* and that deletions/mutations of *USP9Y* can be transmitted from one generation to the next. Here, we generated stable iPSC lines with *AZFa* deletions, tested complementation via introduction of *DDX3Y,* and assessed ability to form germ cells *in vivo* in a xenotransplantation model. We observed a quantifiable improvement in formation of germ cell like cells (GCLCs) from complemented donor iPSCs. Moreover, expression of UTF1, a prospermatogonial protein, was restored in cells complemented by introduction of *DDX3Y* on the *AZFa* background. Whole-genome RNA sequencing of purified GCLCs revealed an enrichment of genes involved in translational suppression and transcriptional control in *DDX3Y*-rescued GCLCs over mutant GCLCs, which maintained a molecular phenotype more similar to undifferentiated iPSCs. This study demonstrates the ability to probe fundamental genetics of human germ cell formation by complementation and indicates that *DDX3Y* functions in the earliest stages of human germ cell development.

Infertility is common, affecting approximately 10–15% of couples with half of all cases involving a male factor^1^,^2^. The most common molecularly-defined cause of male infertility, characterized by production of few or no sperm, is the deletion of one or more *AZoospermia Factor (AZF)* regions of the Y chromosome^3^,^4^,^5^. Deletion analysis of the Y chromosome has revealed three common deletions that are termed *AZFa, AZFb,* and *AZFc* with deletions linked to meiotic recombination errors in highly repetitive sequences within and adjacent to the deletion intervals^6^,^7^,^8^.

Of the three regions, deletions of the *AZFa* region appear to have the most severe outcome for spermatogenesis in men, consistently resulting in a complete absence of spermatogenic cells and a clinical diagnosis termed Sertoli-Cell Only (SCO) syndrome^9^,^10^,^11^. It is generally hypothesized that men with *AZFa* deletions either cannot form or maintain their nascent gem cell populations during or after adolescence^9^. Interestingly, the *AZFa* region contains only two genes, *DBY,* also known as *DEAD Box Helicase 3, Y-linked* (*DDX3Y*) and *Ubiquitin Specific Peptidase 9, Y-linked* (*USP9Y*). While *USP9Y* was originally thought to be a candidate for male fertility, transmittable mutations in *USP9Y* (including a deletion) have been recovered in fertile patients—indicating that *USP9Y* is unlikely to be required for fertility^11^,^12^. The other gene in the interval, *DDX3Y*, remains a viable candidate for fertility^5^,^11^,^13^. *DDX3Y* encodes an ATP-dependent RNA helicase that is a member of the well-conserved *DDX3 DEAD Box Helicase* family that functions in RNA metabolism and translational regulation^14^,^15^,^16^. *DDX3Y*, like many Y-chromosome genes, has a homologue on the X-chromosome, *DBX* or *DDX3X*, with which it shares 91.7% homology^17^. While both *DDX3Y* and *DDX3X* transcripts are expressed widely, in the germ line, DDX3Y protein is restricted to pre-meiotic spermatogonia while DDX3X is expressed in post-meiotic spermatids^10^,^18^,^17^. Thus, it is presumed that DDX3Y protein function has diverged from DDX3X function in regulating germ cell development, and that its deletion is the primary factor responsible for azoospermia in men with *AZFa* deletions^13^,^17^,^18^.

In order to understand the function of *DDX3Y*, we used a previously established xenotransplantation model to examine germ cell formation and probe the genetics of *AZFa* deletions by deriving induced pluripotent stem cells (iPSCs) from men with deletions^19^,^20^. We have previously reported quantitative and qualitative differences in germ-cell like cell (GCLC) formation that phenocopied clinical pathology^19^. Most notably, we demonstrated that *AZFa*-deleted iPSC lines formed the fewest germ cells *in vivo* and displayed differences in mRNA and protein expression relative to iPSCs from fertile men. Here, we introduced the *DDX3Y* gene into the *AZFa*-deleted iPSC line and characterized and compared germ cell development in complemented iPSC lines.

## Results

### DDX3Y is expressed in human fetal germ cells

**Figure 1:**
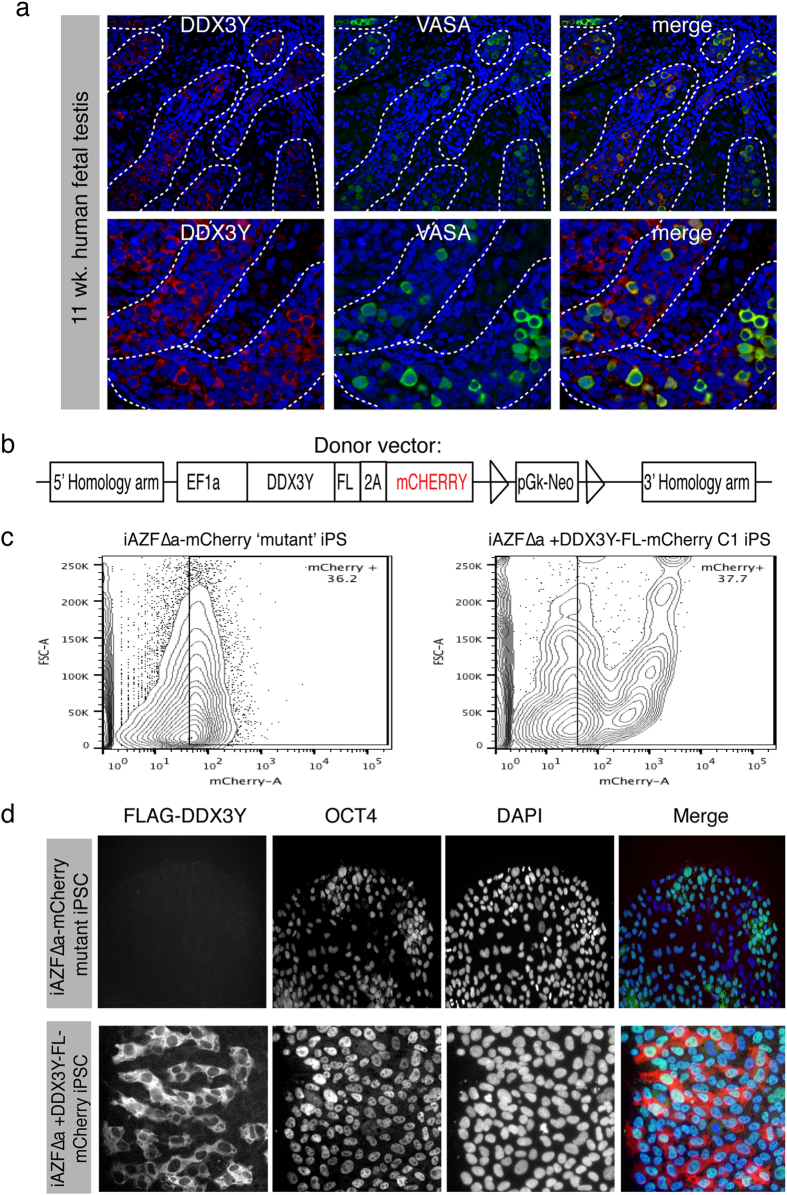
Endogenous expression of DDX3Y and targeting of AZFa-deleted iPSCs. (**a**) Endogenous expression of Ddx3y protein (red) in 11-week-old human fetal testis. Expression of Vasa protein (green) is shown and nuclei are counterstained with DAPI (blue). (**b**) Targeting DNA construct used for homologous recombination of EF1α-driven DDX3Y conjugated to Flag and mCHERRY coding sequences. (**c**) Flow cytometric analysis of mCherry protein expression in targeted iPS cells. (**d**) Immunocytochemical analysis of Flag-Ddx3y and Oct4 protein expression in targeted iPS cells and layered into a merged image (right). Nuclei are counterstained with DAPI (blue).

We began by profiling DDX3Y protein expression in human fetal and adult testes. DDX3Y protein expression is observed in human prospermatogonia during the second and third trimester of fetal testis development^10^,^13^,^18^. We hypothesized that DDX3Y would be expressed in gonocytes or prospermatogonia during the first trimester and therefore explored DDX3Y protein expression in 11 week-old human fetal testes when primitive testis cords enclosing germ cells are discernible. Numerous DDX3Y-positive germ cells were detected in the majority of cells in the tubules (Fig. 1a) in conjunction with expression of the germ cell specific protein, VASA. No DDX3Y expression was observed outside the tubules confirming that the expression was restricted to germ cells, even at such an early developmental stage.

### Genetic complementation of AZFΔa iPSCs with DDX3Y

To genetically complement the *AZFa* deletion, we used TALE Nuclease (TALEN) mediated delivery of a gene construct containing full-length DDX3Y cDNA into iPSCs that harbor an *AZFa* deletion (iAZFΔa). As described in Materials and Methods, we designed a construct (Fig. 1b, Supplementary Fig. 1) that would integrate into the *AAVS* locus of chromosome 19, a region that is often used for integration as it was previously demonstrated to be a “safe harbor” (or expressed constitutively) in gene knock-ins^20^. Our construct contained 5′ and 3′ regions homologous to the *AAVS* locus flanking an *EF1α-DDX3Y-Flag-T2A-mCherry* or an *EF1α-mCherry* insert. A sequence coding for FLAG protein tag (DYKDDDDK) was introduced for immunohistochemical analysis of DDX3Y while Cherry protein expression enabled tracking of targeted iPSC fate *in vitro* and *in vivo.* iAZFΔa cells were then electroporated with a construct containing either *EF1α-mCherry* or *EF1α-DDX3Y-Flag-T2A-mCherry*. Following gene delivery and antibiotic selection, stable iAZFΔa clonal cell lines that expressed DDX3Y-FLAG and Cherry proteins were isolated. Note that we selected lines that expressed *DDX3Y* constitutively and found that integration into the *AAVS* locus was not required for constitutive expression. PCR and sequence analysis confirmed that the entire EF1α-DDX3Y-Flag-T2A-mCherry construct was inserted in full into the genome (Supplementary Fig. 1b). To purify positively targeted cells from the entire population, we used cell sorting (Fig. 1c) to recover all Cherry+ cells, which were subsequently expanded under standard conditions. Each rescued or mutant clonal line continued to express DDX3Y-Flag and Cherry after several weeks of serial passaging and routine maintenance *in vitro*. Moreover, overexpression of the *DDX3Y* construct did not alter stem cell identity, as both *DDX3Y*-rescued and mutant clones maintained normal stem cell morphology and expressed markers of pluripotency (Fig. 1d).

### DDX3Y overexpression does not improve engraftment efficiency but favors germ cell formation compared to mutant iAZFΔa line

**Figure 2:**
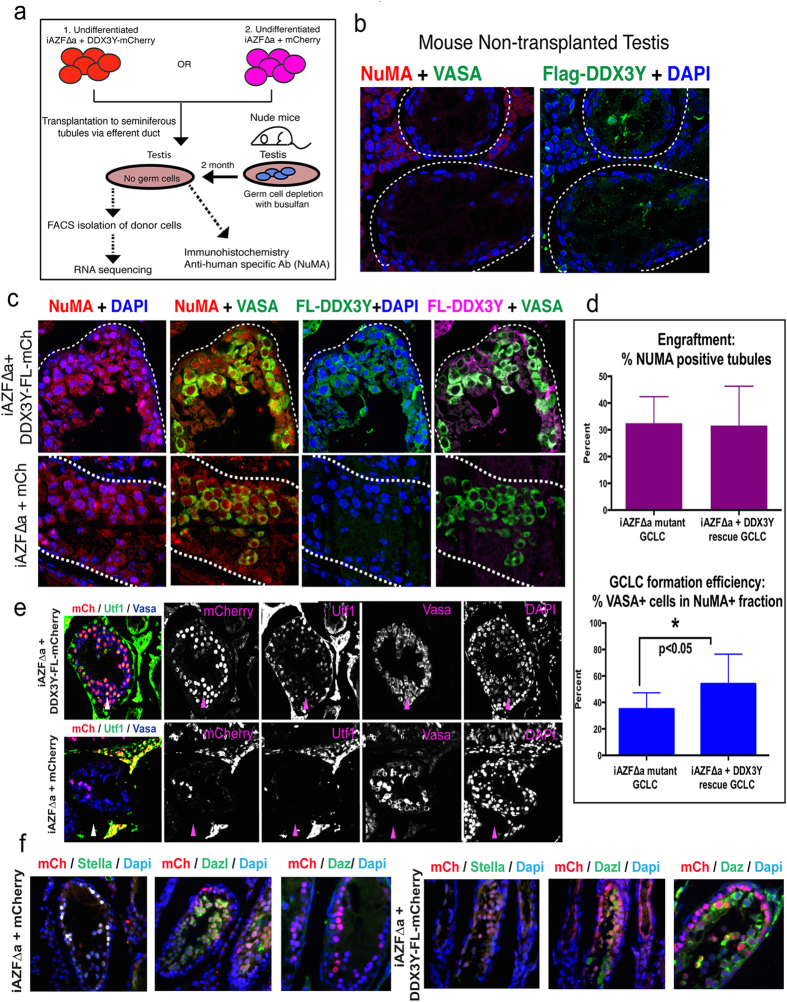
Germ cell-like cell formation from targeted iPSCs following xenotransplantation to mouse seminiferous tubules. (**a**) Experimental scheme for xenotransplantation experiments, purification and downstream analysis of donor-derived cells. (**b**) Testicular expression of NuMA (red) and Flag-Ddx3y (green) in non-xenotransplanted, busulfan-treated mice. (**c**) Expression of NuMA (red) and Flag-Ddx3y (green) in xenotransplanted seminiferous tubules. NuMA and Flag-Ddx3y are co-stained with Vasa and nuclei are counter-stained with DAPI (blue). Top row is DDX3Y-rescued donor cells and bottom row, mutant GCLCs. (**d**) Top, percentage efficiency in engraftment of NuMA+ donor cells. Bottom, percentage of NuMA+/Vasa+ cells in seminiferous tubules of testis transplanted with both DDX3Y-rescued iPSCs and *AZFa*-deleted mutant iPSCs. Totally, 3 independent xenografts were quantified per donor cell line. (**e**) Detection of Utf1, Cherry and Vasa proteins in xenotransplants from rescue (iAZFΔa+DDX3Y-FL-mCherry) and mutant (iAZFΔa+mCherry) lines. A merged image of all three channels and DAPI-stained nuclei (blue) is accompanied by individual images of each protein and DAPI in gray scale. (**f**) Detection of Cherry, Stella, Dazl and Daz proteins in xenotransplants from rescue (iAZFΔa+DDX3Y-FL-mCherry) to the right and mutant (iAZFΔa+mCherry) lines to the left of the panel. Merged images are shown with DAPI-stained nuclei in blue.

To assess the effect of restoring DDX3Y on germ cell formation from iPSCs, we used xenotransplantation with two clones of undifferentiated iPSCs from either the rescue lines (iAZFΔa+DDX3Y-mCherry) or the mutant lines (iAZFΔa+mCherry) into mouse seminiferous tubules (Fig. 2a). Xenotransplantation of human stem cells into mouse seminiferous tubules has been used to test germ cell differentiation from human spermatogonial stem cells and from pluripotent stem cells, thereby allowing one to assess the potential of genetically distinct lines to form germ cell-like cells (GCLCs)^19^,^21^,^22^,^23^. Testes of non-xenografted, busulfan-treated mouse recipients were negative for NuMA, VASA or DDX3Y-FLAG (Fig. 2b). Two months post-transplantation, six individual testis xenografts per cell line were analyzed by immunohistochemistry using tissue cross-sections to locate donor-derived GCLCs positive for the human cell-specific protein, NuMA (Fig. 2c). NuMA+ GCLCs were then co-labeled with VASA and an antibody against FLAG to identify DDX3Y-FLAG proteins. All NuMA+ GCLCs from the rescue line were DDX3Y-FLAG positive and the majority of DDX3Y-FLAG+ GCLCs appeared to be VASA positive. As expected, the mutant donor line did not have cells positive for DDX3Y-FLAG (Fig. 2c, bottom row). We then determined the efficiency of donor cell engraftment and of GCLC formation from donor cells. For this purpose, we counted the number of tubules containing NuMA+ cells across several tissue cross-sections for each xenograft as described in Materials and Methods); we observed that there was no significant difference in the number of engrafted tubules between rescue and mutant lines (Fig. 2d). However, in contrast, tubules that were transplanted with the rescue line contained a higher percentage of VASA+ GCLCs (Fig. 2d). On average, approximately 55% of NuMA positive cells in the tubule were also positive for the germ cell marker VASA, compared with only 38% of NuMA and VASA double positive GCLCs in tubules transplanted with the mutant lines.

### Donor-derived cells in murine seminiferous tubules express germline proteins

To better stage and assess the relative quality of the germ cells derived from *AZFa* mutant and rescue iPSCs, we used immunohistochemistry to stain for the presence of various germ cell-specific proteins. In addition to the germ cell marker VASA, the PGC/gonocyte stage markers DPPA3 (also known as STELLAR) and DAZL were expressed in mCherry+ GCLCs from both *AZFa* mutant and rescued recipient testes (Fig. 2f). DAZ1 and UTF1 spermatogonial proteins were never detected in mouse seminiferous tubules transplanted with AZFΔa mutant cells (two independent testis xenografts were checked for each). In contrast, both UTF1 and DAZ1 protein were detected in a subset of mCherry+ DDX3Y-rescued iAZFΔa GCLCs (Fig. 2e). For all primary antibodies used in this study for immunostaining, the corresponding isotype IgG antibodies were used to stain xenograft tissue sections cut from the same tissue block. Only low or no background isotype IgG antibody signals were broadly observed across all four antibodies tested (Supplementary Fig. 2).

**Figure 3:**
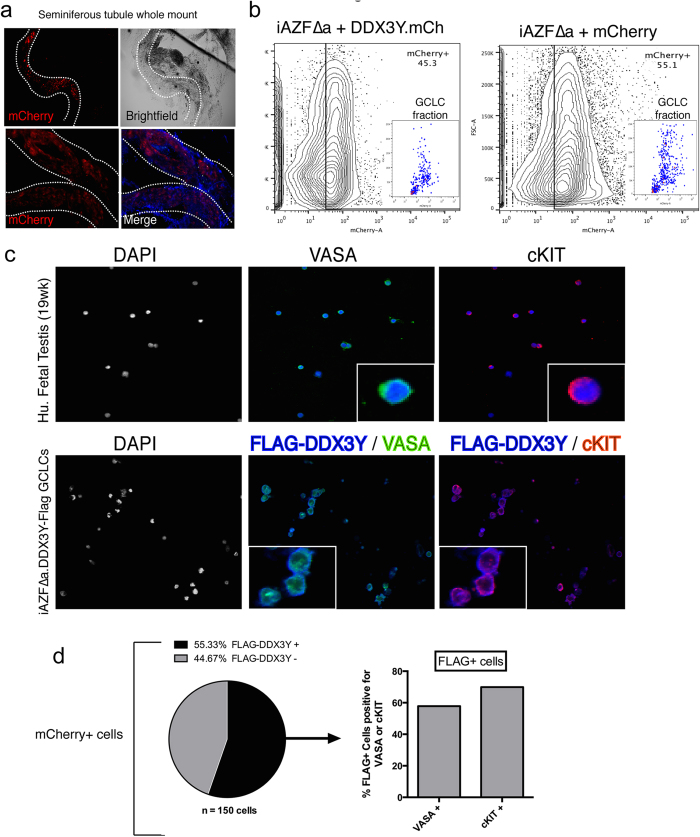
Purification and analysis of donor-derived GCLCs from seminiferous tubules of xenografts. (**a**) Whole mount imaging of mCherry+ donor-derived cells inside seminiferous tubules following 2 months of xenotransplantation. (**b**) Flow cytometric analysis and enrichment of mCherry+ GCLCs extracted from seminiferous tubule by enzymatic digestion. Inset, purity analysis of mCherry+ cells after FACS-based purification. (**c**) Immunocytochemical analysis of GCLCs purified from xenografts transplanted with DDX3Y-rescued iPSCs. For comparison, fetal germ cells purified from 19-week-old human fetal testis were also immunostained. Vasa (green) , Flag-Ddx3y (blue) and cKIT (red) were both stained. Nuclei were counter-stained with DAPI (blue) where indicated. (**d**) From the fraction of mCherry+ cells sorted from FACs, the percentage of Flag-Ddx3y+ cells that co-expressed either Vasa or cKIT was determined by immunocytochemistry and expressed in a bar chart.

In parallel with immunohistochemistry, we also developed a strategy to purify donor-derived GCLCs from mouse xenografts. Two months post-transplantation, testis xenografts derived from mutant (iAZFΔa+mCherry) and DDX3Y-rescue (iAZFΔa+DDX3Y-mCherry) lines were harvested (two testes from two mice per line) and dissociated with a two-step enzymatic treatment that was adopted and modified from published methods^22^. Prior to enzymatic dissociation, we verified the presence of mCherry+ donor cells inside seminiferous tubules using whole mount imaging (Fig 3a). Subsequently, the resultant germ cell fraction was analyzed by flow cytometry and all donor cells positive for mCherry (approximately half the population of viable, gated cells for each line) were sorted out from the entire fraction (Fig. 3b). Sorted GCLC fractions were allocated into 2 fractions for immunocytochemical analysis and RNA extraction, respectively. We first measured the expression of the germ cell protein VASA, and the pro-spermatogonial protein, cKIT, in purified GCLCs derived from the rescue line. In agreement with the cross-sectional immunohistochemical analyses and with germ cells from the human fetal testis (Fig. 3c), >50% of the intratubular cells were of donor origin (~55% DDX3Y-FLAG+) and VASA or cKIT were expressed in roughly half each of the DDX3Y-FLAG+ population (Fig. 3d).

### DDX3Y-complemented GCLCs exhibit a divergent transcriptome from non-complemented GCLCs and iPSCs

**Figure 4:**
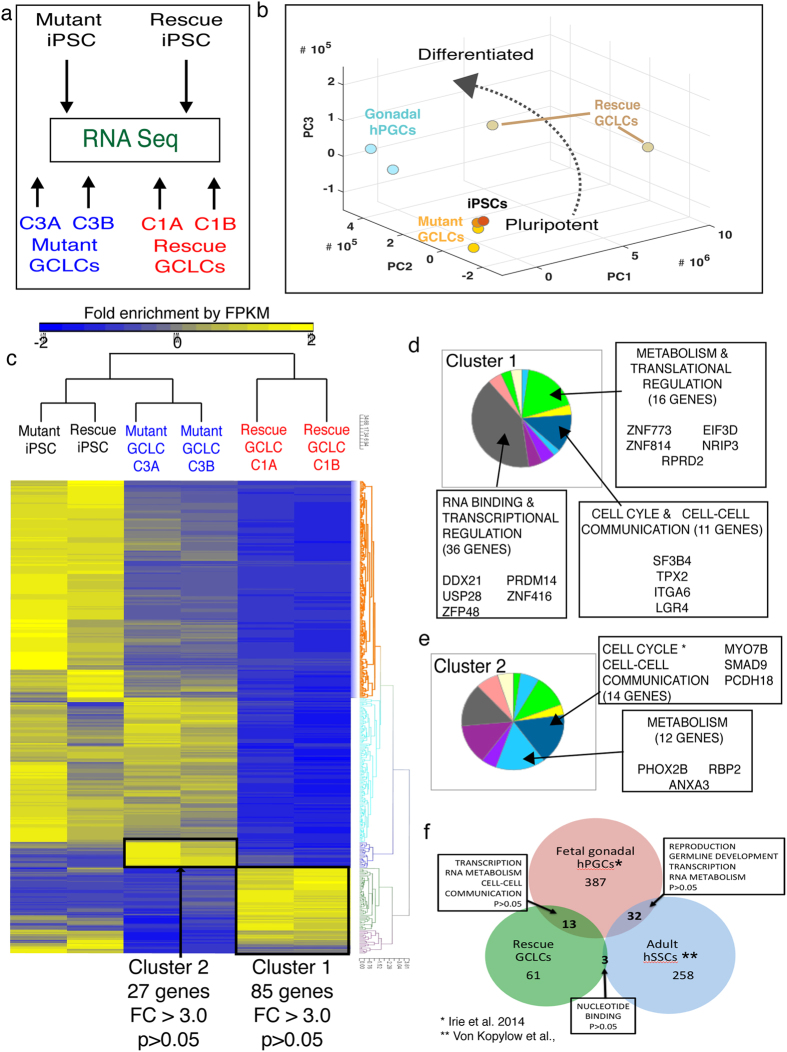
Global gene expression analysis of GCLCs by RNA sequencing and correlation with human endogenous germ cells. (**a**) Schematic of RNA sequencing analysis in two donor iPSC lines and two xenograft replicates each of *AZFa*-deleted mutant GCLCs (mutant GCLCs) and DDX3Y-rescue GCLCs (rescue GCLCs). (**b**) Three-dimensional (3D) Principal Component Analysis (3D-PCA) of global transcript levels in donor iPSCs, mutant GCLCs, rescue GCLCs and human gonadal PGCs (week 7-old embryo). (**c**) Hierarchically clustered heat map displaying fold enrichment of all differentially expressed transcripts between the 6 samples after RNA sequencing. Clusters 1 and 2 indicate regions of interest that were submitted for GO analysis in (D). (**d**) Gene Ontology (GO) analysis by PantherDb software of cluster 1, genes up regulated in two replicates of rescue GCLCs versus two replicates of mutant GCLCs. GO categories are summarized in a pie chart and call-out boxes highlight genes of interest. All genes were up regulated by fold changes >3.0. (**e**) GO analysis by PantherDb software of cluster 2, genes up regulated in two replicates of mutant GCLCs versus two replicates of rescue GCLCs. GO categories are summarized in a pie chart and genes of interest are highlighted by call-out boxes. (**f**) Venn diagram representing relationship between genes up regulated by fold change of 2.0 or greater and with significance *p* < 0.05 in cluster 1 of rescue GCLCs, embryonic PGCs (Irie *et al.*, 2015) and adult SSCs (Von Kopylow *et al.*, 2010). Overlapping genes in various segments of Venn diagram were categorized by GO analysis using PantherDb and indicated in call-out boxes.

We then analyzed global transcription in *DDX3Y*-rescued GCLCs and compared it to that of AZFΔa mutant GCLCs (Fig. 4a). To achieve this, we performed whole-genome RNA sequencing of GCLCs purified from the xenografts of DDX3Y-rescue iPSCs as well as AZFΔa mutant iPSCs. Raw RNA sequencing reads were subjected to a standardized bioinformatic workflow in order to filter in reads mapping to the human genome^23^. By three-dimensional principal component analyses, PCA, (Fig. 4b), we compared the relationships between different cell types using whole-genome filtered expression data derived from the iPSC donor lines, two independent xenografts of the rescue iPSC line (iAZFΔa+DDX3Y-mCherry C1A & C1B) and two xenografts of the mutant iPSC line (iAZFΔa+mCherry C3A and C3B). We also included whole-genome RNA sequencing datasets (2 biological replicates) published by Irie *et al.* that were derived from week 7-old human gonadal PGCs (hPGCs)^24^. PCA analysis demonstrates that DDX3Y-rescued GCLCs, C1A and C1B, were significantly different in transcription profiles from the mutant GCLCs which exhibited very close similarity to donor iPSCs in PC1, PC2 and PC3 axes. Meanwhile, hPGCs occupied a discrete position in the 3D-PCA but aligned closely in PC2 and PC3 axes, at least in part, with two replicates of rescue GCLCs (Fig. 4b). Pairwise volcano plots emphasize the gene expression differences between each sample (Supplementary Fig. 3a). The Jensen-Shannon distance between rescue GCLCs, mutant GCLCs and donor iPSCs was also indicative of the divergence in global transcript enrichment between iPSCs and GCLCs as well as between rescue and mutant GCLCs (Supplementary Fig. 3b). We further explored the relationship between the 2 GCLC populations (pooled among xenograft replicates) and the 2 donor iPSC lines by representing differential mRNA expression in a hierarchically clustered heatmap (p < 0.05) matrix (Fig. 4c). Approximately 248 transcripts (p < 0.05) were differentially expressed between iPSCs, rescue GCLCs and mutant GCLCs. Cluster 1 represents 85 genes enriched in rescue GCLCs over mutant GCLCs, while cluster 2 represented transcripts enriched in mutant GCLCs over rescue GCLCs (Fig. 4c & Supplementary Table 1). Most strikingly, a large number of transcripts that were abundant in iPSCs and mutant GCLCs were absent or significantly down regulated in rescue GCLCs. A subset of pluripotency genes including POU5F1, SOX2 and NANOG were hierarchically clustered in a heatmap and we noted that mutant GCLCs were substantially enriched in expression of pluripotency genes (similar to donor iPSCs) relative to rescue GCLCs (Supplementary Fig. 3c).

### Gene ontology analysis of genes enriched in DDX3Y-complemented GCLCs and relationship with endogenous human germ cells

We next categorized genes in clusters 1 and 2 (Fig. 4c) via Gene Ontology (GO) categories^25^ (Fig. 4d & Supplementary Table 1 & 2). A large number of genes up regulated in the rescue GCLCs encode zinc-finger transcription factors and RNA-metabolism regulators including ZFP48, ZNF578, DDX21 and USP28 (Fig. 4d). Additionally, the known germ cell regulators, NANOG, LIN28A and PRDM14 were enriched in rescue GCLCs. In mutant GCLCs, up regulated genes included a handful of transcription factors but no RNA metabolism genes (Fig. 4e). Both mutant and rescue GCLCs did express a handful of cell cycle regulatory genes. We then compared the set of genes up regulated in rescue GCLCs to two recently published gene lists: 1) A list derived from human fetal PGCs (hPGCs) in week 7 embryos^24^ and 2) a list generated from adult human spermatogonial stem cells (hSSCs)^26^. We investigated the relationships of the three gene lists by plotting a Venn diagram and observed that rescue GCLCs shared more genes in common with hPGCs than hSSCs, as we predicted (Fig. 4f; Supplementary Fig. 2d). Overall, gene ontology analysis by biological process revealed common enrichment in 13 genes in hPGCs and rescue GCLCs genes involved in transcription and RNA metabolism including PRDM14, RAB39A, and ARID3B among others. In contrast, only 3 genes involved in nucleotide binding were commonly enriched in rescue GCLCs and hSSCs. As we predicted, hPGCs and hSSCs shared genes involved in gamete development, gonadal development, transcription and RNA metabolism, all processes involved in germ cell development.

## Discussion

Murine seminiferous tubules are able to induce germ cell formation from *AZFa*-deleted iPSCs, thus enabling studies to examine the function of *AZFa* genes in human germ cell development. Here, we observed two significant differences in germ cell formation in *AZFa-*deleted cells relative to cells that were complemented with *DDX3Y*: first, we observed quantitative enhancement in GCLC formation and robust expression of human PGC proteins in all complemented lines, as well as a modest expression of two spermatogonial lineage proteins in rescued GCLCs. Second, we observed that *DDX3Y*-complemented GCLCs activate a transcriptional program that is enriched in translational modulators and transcriptional repressors. Most strikingly, rescued GCLCs exhibited a divergent transcriptomic fate from non-complemented GCLCs that was closer to that of PGCs and prospermatogonia. In contrast, mutant cells maintained a transcriptional profile more similar to undifferentiated iPSCs.

Previous work suggested that *DDX3Y* encodes an early regulator of spermatogonial development and may control downstream differentiation of male gametes^11^,^13^. Our results offer a glimpse into DDX3Y function during the developmental phase spanning PGC specification to prospermatogonial development in humans. Our data indicate that gene rescue of the *AZFa* deletion is sufficient to restore early germ cell formation in *AZFa*-deleted iPSCs, at least in part, along with restoration of the expression of a subset of genes synonymous with embryonic germline development including PRDM14. In addition, in previous studies^19^, we observed that a subset of GCLCs derived from iPSCs derived from fertile controls expressed spermatogonial proteins such as UTF1, DAZ, SALL4 and PLZF. Therefore, we hypothesized that DDX3Y-rescued iPSCs would exhibit differentiation properties consistent with AZF-intact iPSCs in the xenotransplantation model. In line with this, at the protein level, DAZ and UTF1 proteins were exclusively detected only in a subset of DDX3Y-rescued GCLCs. Although their expression is not widespread in GCLCs, the presence of these two germ cell-specific proteins suggests that DDX3Y-rescued GCLCs have progressed to the prospermatogonial cell stage and that DDX3Y may regulate this early transition. We note, however, that we did not detect other later-stage spermatogonial protein markers such as PLZF, GFRα1 or cKIT which supports the notion that DDX3Y-rescued GCLCs only differentiate to the prospermatogonial stages of germline development in the mouse xenograft system.

Of additional interest was the increase in expression of translational and transcriptional modulator genes with complementation of *AZFa* deletions with *DDX3Y*. Although the precise molecular mechanisms will need to be explored further, our findings bolster the mounting evidence that indicates that RNA-binding proteins and in particular, DEAD-box RNA helicases act as key regulators of regulating male germline development^16^,^17^,^27^,^28^,^29^,^30^. We suggest two potential models for *DDX3Y* function in germline development. One is that *DDX3Y* directly activates expression of RNA metabolism genes that have roles in translational and transcriptional regulation. Genes such as *DDX21, KHDRBS3, SF3A1, RAB39A* and *RPRD2* are all highly enriched in rescue GCLCs and a subset of these genes share expression in human fetal PGCs. In addition, whole-genome expression patterns of *DDX3Y*-rescued GCLCs are more aligned with that of gonadal hPGCs than non-rescued GCLCs. These genes play diverse roles in mRNA processing, pre-mRNA splicing and RNA polymerase II regulators respectively^31^,^32^,^33^,^34^,^35^. Since control of translational activity has been shown to be a highly conserved germline function from worms and mice to humans^36^,^37^, one potential mechanism for translational control is via sequestration of mRNA in P-granules and ribonucleoprotein particles (RNPs) in postnatal germ cells^38^. Based on this evidence, we propose that DDX3Y, via its RNA helicase domain, may contribute to translational modulation via binding to small ribosomal RNA or mRNA molecules potentially in cytoplasmic p-granule-like structures.

A second proposed model for DDX3Y is via transcriptional regulation. In mammalian (murine) PGCs entering spermatogonial development, gene-specific transcriptional activity is repressed and is maintained through the activity of conserved zinc-finger transcription factors such as Prdm1(Blimp1) and Prdm14^24^,^39^,^40^,^41^. Curiously, in *DDX3Y*-rescued GCLCs, we observed a strong enrichment of expression of six zinc-finger transcriptional regulators including *ZNF416, ZFP48* and *ZFP42* in addition to *PRDM14*. This data suggests that the rescue by *DDX3Y* may initiate global transcriptional ‘remodeling’ via the action of a cohort of zinc finger transcription factors. Although specific gene targets are not revealed in this study, we suggest that the enriched expression of zinc finger proteins could repress gene expression by recruiting transcriptional co-repressor proteins and enabling repressive histone modifications, in a similar fashion as observed with Blimp1 and Prdm14 proteins in mice^40^,^41^. Our data also supports the possibility of histone remodeling, because of the enrichment of RNF168, an E3 ubiquitin ligase that is linked to histone remodeling^42^. If this concept holds, it may explain the striking decrease in global transcript levels in rescue GCLCs, in contrast to mutant GCLCs and donor iPSCs. Further studies will explore the relationship between DDX3Y and other early RNA-binding proteins such as DAZL, VASA (DDX4) and NANOS3 with known roles in germ cell development. Additionally, direct interactions between DDX3Y protein and pre-mRNA molecules would reveal DDX3Y downstream targets. Overall, we propose a model in which DDX3Y is expressed after human PGC specification occurs and contributes to the transcriptional silence and translational suppression programs of the male germline during pre-meiotic stages of spermatogenesis. Our data does not disprove that alternative molecular pathways exist for early human germ cell formation in both sexes. Instead, we propose that *DDX3Y* is a testis-specific gene, expressed from the *AZF* regions of the Y chromosome, that acts as an early spermatogenesis regulator. Our data indicates that DDX3Y expression is sufficient to drive germ cell differentiation on an *AZFa*-deleted background.

## Materials and Methods

### Study participants

All experimental protocols involving human skin biopsy procedures, human fibroblast derivation, derivation of induced pluripotent stem cells (iPSC) and iPSC culture for research use were approved by the Stanford University Institutional Review Board and the Stanford University Stem Cell Research Oversight (SCRO) committee. All experimental procedures involving animals were approved by the Institutional Animal Care and Use Committee in accordance with their guidelines. Study participants were recruited by obtaining a written informed consent from each individual participant in accordance with Stanford University Institutional Review Board guidelines. Second trimester Human fetal testes were staged and procured from Advanced Bioscience Resources (ABR Inc., Oakland, CA). In total, two 1^st^ trimester and six 2^nd^ trimester human fetal testes were procured for this study and used in xenotransplantation assays or for immunohistochemistry.

### Construct Design

The design of a DDX3Y gene delivery construct was executed as follows and described in Supplementary Methods. First, DDX3Y cDNA was amplified out from Image Clone #BC034942.2 (Thermo Fisher Scientific) using the following primer pair: Forward primer (5′GATCCGGCCGCCTCGGCCGCCGCCACCATGGATTACAAGGATGACGACGATAAGAGTCATGTGGTGGTGAA containing a 5′ SfiI site) and a reverse primer (5′ CCCTCTGAGACCACCGGTCCCTTATCGTCGTCATCCTTGTAATCGTTGCCCCACCAGTCA containing a 5′ Flag tag site) and subcloned into OneShot TOP10 cells (Thermo Fisher Scientific). A blunt-end fragment containing DDX3Y-Flag-T2A-mCherry was then PCR amplified using the forward primer 5′ cacc atg AGTCATGTGGTGGTGAA 3′ and a reverse primer 5′ CTACTTGTACAGCTCGTCCA 3′. This PCR product was then ligated into the pENTR/D TOPO vector using the pENTR Directional Cloning Kit (Thermo Fisher Scientific). The pENTR-DDX3Y-FLAG-T2A-mCherry was then recombined using the Gateway LR recombination method with an EF1-alpha promoter-containing pENTR entry vector and a p2K7 destination vector containing ~500 bp long 5′ and 3′ homology arms to the AAVS locus of Chromosome 19. Following recombination, the resultant product was transformed into TOP10 chemically competent cells and successful recombination events were screened for using ampicillin and blue/white colony selection.

### Construction of DDX3Y-expressing iPSC Lines

Ten μg of AAVS-EF1alpha-DDX3Y-FLAG-mCHERRY-AAVS (or the AAVS-EF1alpha-mCHERRY empty vector), and 2.5 μg of each AAVS TALEN were electroporated using the Neon Transfection System (Thermo Fisher Scientific) into 1–1.5 million cells from two independent *AZFΔa* iPSC cell lines derived from a single patient donor. Following electroporation, single cell suspensions were plated onto Matrigel-coated 10 cm tissue-culture dishes at a density of 250,000 to 500,000 cells per dish. After a 24 hour period of cell attachment, positively electroporated iPS cells were selected in mTeSR growth medium supplemented with 50ng–100ng/uL of geneticin (G418) for two weeks. Each individual surviving colony was manually passaged into 48 well plates and expanded. DNA was collected using the QuickExtract DNA Extraction Solution (Epicenter). Colonies were screened for site-specific integration using primers internal to the AAVS locus and primers internal to the construct only (see Supplementary Fig. S1). Out of 101 colonies screened, none had AAVS locus-specific integrations. However, 3 colonies from EF1a-DDX3Y-FLAG-mCHERRY clones and 2 colonies from EF1a-mCHERRY showed stable mCherry expression for over 5–6 passages. When sequenced, all 5 clones were positive for targeting of the respective construct. These 5 colonies were grown, expanded and used for all future studies.

### Xenotransplantation Assay

Human cell lines were transplanted into the testes of busulfan-treated, immune-deficient nude mice (NCr nu/nu; Taconic) as previously described for primate and human spermatogonia (Ramathal *et al.*, 2014, Hermann *et al.*, 2010). Two clonal cell lines each of the iAZFΔa-DDX3Y-Flag-mCherry, or the control line iAZFΔa-mCherry were transplanted. Briefly, immunodeficient nude mice were treated with a single dose of busulfan (40 mg/kg, Sigma) at 6 weeks of age to eliminate endogenous spermatogenesis. Xenotransplantation was then performed five weeks after busulfan treatment by injecting 7–8 μl cell suspensions containing 500,000–600,000 cells total (either iAZFΔa-DDX3Y-Flag-mCherry or iAZFΔa-mCherry lines), or a mixture of equal parts of both lines. Three mice were xenotransplanted in total per cell line (i.e. six testes in total per cell line). All cell suspensions contained 10% trypan blue (Thermo Fisher Scientific) and were directly injected into the seminiferous tubules of each recipient testis via cannulation of the efferent ducts. Eight weeks after transplantation, recipient mouse testes were harvested for donor cell isolation, whole-mount immunostaining and whole testes immunohistochemical analyses.

### Donor-cell isolation from spermatogonial tubules

The tunica layer of xenotransplanted testicular tissues was gently peeled off and the entire tissue was teased apart. To dissociate interstitial compartments from spermatogonial tubules an enzyme solution containing Collagenase type IV (1 mg/mL) and DNase I (7 mg/mL) in Hank’s Balance Salt Solution (HBSS) was used at room temperature. The cell and tubular mixture was subsequently passed through a cell sieve to separate the interstitial fraction from tubules. Next, the tubules were washed in HBSS and then incubated in an enzyme mixture containing Trypsin-EDTA (0.25%) and DNase I (7 mg/mL) at 37 °C to release all germ cells and intratubular cells from the tubules into cell suspension. For all enzyme steps, the tissues were agitated with pipetting intermittently. The final incubation was neutralized with HBSS supplemented with 10% FBS and the final cell fraction was sieved through a 100 μm cell sieve. The resultant cellular mixture was counted and cell stocks made for cryopreservation or for FACS-based analysis. For whole-mount visualization of donor mCherry-positive cells inside tubules, the dissociated seminiferous tubules were washed and mounted with VectaShield mounting media containing DAPI (Vector Laboratories) under raised coverslips and imaged with fluorescent confocal microscopy.

### Fluorescence-activated cell sorting (FACS) of donor-derived cells from testis xenotransplants

Donor-derived cells purified from spermatogonial tubules were counted and divided into fractions for cell FACS analysis. Approximately 1–1.2 million cells were harvested from each of 2 xenotransplanted testes per cell line. The entire intratubular fraction of cells from each testis was individually analyzed on a Becton Dickinson FACs Aria II cell sorter. Sorting gates were established based on forward and side scatter as well as the level of mCherry expression after exclusion of dead cells or debris stained with DAPI. Cells positive for mCherry expression were directly sorted into a PBS supplemented with 10% FBS. The sorted fraction was reanalyzed on the sorter for purity determination. Subsequently, 90% of the sorted cells were pelleted and resuspended in PicoPure RNA extraction buffer (Thermo Fisher Scientific) for RNA analysis by sequencing. The remaining 10% of cells were resuspended in PBS, dropped onto glass slides and then fixed in 20% ice-cold methanol. Following fixation, cells were washed and rehydrated in PBS for immunocytochemical analysis.

### Immunohistochemistry

Formalin-fixed mouse testes xenotransplants were paraffin embedded and sectioned into serial cross-sections of 5–10 μm thickness each (AML Laboratories). Testis sections were deparaffinized in xylene, rehydrated through an ethanol-graded series. For all samples, antigen retrieval was performed by boiling the sections in 0.01 M sodium citrate buffer (pH 6.0) for 20 min, followed by incubation at room temperature for 30 min. A 10% solution of normal donkey serum (Jackson ImmunoResearch) in PBS was used as a blocking buffer. Sections were incubated with the following primary antibodies diluted in blocking solution (1.0% Normal Donkey Serum, 0.1% Triton X-100, and sterile PBS) overnight at 4 °C: VASA (1:200) and GFRα1 (1:250) (R&D Systems); NuMA (1:200), STELLA (1:200), DAZL (1:200), and DAZ (1:250) (Abcam); UTF1 (1:200; Millipore); PLZF (1:250; Chemicon); OCT4 (1:500), SOX2 (1:500). The sections were washed and labeled with Alexa dye-conjugated secondary antibodies. Sections were mounted in ProLong Gold Antifade mounting media containing DAPI (Life Technologies). Negative controls included incubation with rabbit immunoglobulin G antibodies and omission of the primary antibody for all samples. Quantification of sections for NuMA/VASA double staining was determined manually from three to five independent 20× fields taken from three different testis tissue depths and from at least three separate biological replicates. Data for statistical analysis follow a normal distribution.

### Whole Transcriptome RNA Sequencing Analysis

Total RNA was extracted with the PicoPure RNA extraction kit (Thermo Fisher Scientific) per manufacturer’s instructions and subjected to cDNA synthesis. The original donor iPS cell lines (iAZFΔa-DDX3Y-FL-mCherry & iAZFΔa-mCherry) and the entire RNA isolate from each testis was subjected to first and second cDNA synthesis using the Ovation RNA-Seq System V2 (NuGEN Technologies, Inc.; San Carlos, CA) following the fragmentation with an average size of 200–300 bases using the Covaris S-Series System. Briefly, 1 ug of each cDNA sample was diluted into 120 μl 1X TE buffer. The Covaris S-Series System settings were as follows: duty cycle −10%, intensity −5, cycles/burst −100, time –5 min. Illumina library construction was then performed using the NEBNext DNA Sample Prep Master Mix Set 1 and Agencourt AMPure XB beads for clean up. Briefly, 250 ng of fragmented DNA was end repaired following the manufacturer’s instructions of the NEBNext DNA Sample Prep master Mix Set 1 kit followed by cleanup with the Agencourt RNAClean XB beads. End repaired DNA was subject to dA-tailing followed by a second clean up. dA-tailed DNA and adaptor were ligated followed by a PCR for enrichment of adaptor ligated DNA (98 °C for 30 sec; 17 × 98 °C for 10 sec, 65 °C for 30 sec, 72 °C for 30 sec; 72 °C for 5 min, 4 °C on hold). Samples were cleaned once again and the built library was analyzed on a HS Agilent DNA chip using a Bioanalyzer. A total of 6 Samples were sequenced in 1 lane of Illumina HiSeq 2000 platform (Illumina, Inc.) as 100 bp paired end reads. Quality check of raw data was processed through the web-based Galaxy platform using the FASTQC tool. Reads with a median score lower than 20 were trimmed using FASTQ Trimmer. Reads were then mapped using TopHat v.2.0.5 with default settings. The mean insert sizes as determined by the Bioanalyzer were employed in the TopHat mapping. Transcript assembly and expression level quantification of transfrags was performed using Cufflinks v.2.0.2 to filter out background and artifactual transfrags (Trapnell *et al.*, 2012). Each sample was assembled individually and all assemblies were merged together using Cuffmerge. Bowtie indexes and annotation files were downloaded from http://cufflinks.cbcb.umd.edu/igenomes.html (UCSC, h19). Transcripts with a p < 0.05 were considered to be differentially expressed. Visualization of differential gene expression analysis was performed with CummeRbund v.1.2.0 and Panther Gene Expression Suite analysis. Principal component analysis was computed using the cummeRbund package in R (version 3.1.2). Matlab (version 8.4.0) was used to visualize the projection for all three principal components.

### Statistical Analysis

Quantification of sections for NuMA/VASA double staining was determined manually from five to six independent 20× fields taken from three different testis tissue depths and from at least three separate biological replicates (individual xenografts). Data for statistical analysis follow a normal distribution. Quantification of immunocytochemical staining in dropped cells was performed manually from ten independent 40x fields taken from each cell population. Analysis of variance (ANOVA) statistical comparisons were performed using GraphPad Prism (La Jolla, CA) with statistical significance set at no higher than α = 0.05. For FACS analysis of xenografts, mCherry-positive cellular fractions (%) were determined from two testes and the average result of the two is shown along with a representative image. Principal component analysis was computed using the cummeRbund package in R (version 3.1.2). Matlab (version 8.4.0) was used to visualize the projection for all three principal components. Raw RNA sequencing data from the manuscript by Irie *et al.* were downloaded from the GEO Accession viewer and processed in an identical manner as described above (http://www.ncbi.nlm.nih.gov/geo/query/acc.cgi?acc=GSE60138). For analysis of differentially expressed gene clusters from RNA sequencing data, PantherDb software was used (http://www.pantherdb.org) to generate pie charts and filter Gene Ontology (GO) categories. BioVenn web-based application (http://www.cmbi.ru.nl/cdd/biovenn) was used to calculate overlap of genes and transcripts between cluster 1 (transcripts with fold change >2.0 and *p* > 0.05) of the RNA sequencing datasets obtained from this study and the two referenced publications to generate a Venn diagram^43^.

## Additional Information

**How to cite this article**: Ramathal, C. *et al.* *DDX3Y* gene rescue of a Y chromosome *AZFa* deletion restores germ cell formation and transcriptional programs. *Sci. Rep.* **5**, 15041; doi: 10.1038/srep15041 (2015).

## Supplementary Material

Supplementary Information
