# Supplementary material for: DDX3Y gene rescue of a Y chromosome AZFa deletion restores germ cell formation and transcriptional programs
Source: Sci Rep. 2015 Oct 12;5:15041. doi: 10.1038/srep15041 (PMC4601010; doi:10.1038/srep15041)
Supplement: Supplementary Information [file srep15041-s1.pdf]

Supplementary Information

DDX3Y gene rescue of a Y chromosome *AZFa* deletion restores germ cell formation and transcriptional programs

Cyril Ramathal[1,*], Ben Angulo[1,2,*], Meena Sukhwani[3], Jun Cui[1,2], Jens Durruthy Durruthy[1], Fang Fang[1,2], Paula Schanes[1], Paul J. Turek[3], Kyle E. Orwig[4], & Renee Reijo Pera[1,2 **]

Supplementary Figure 1

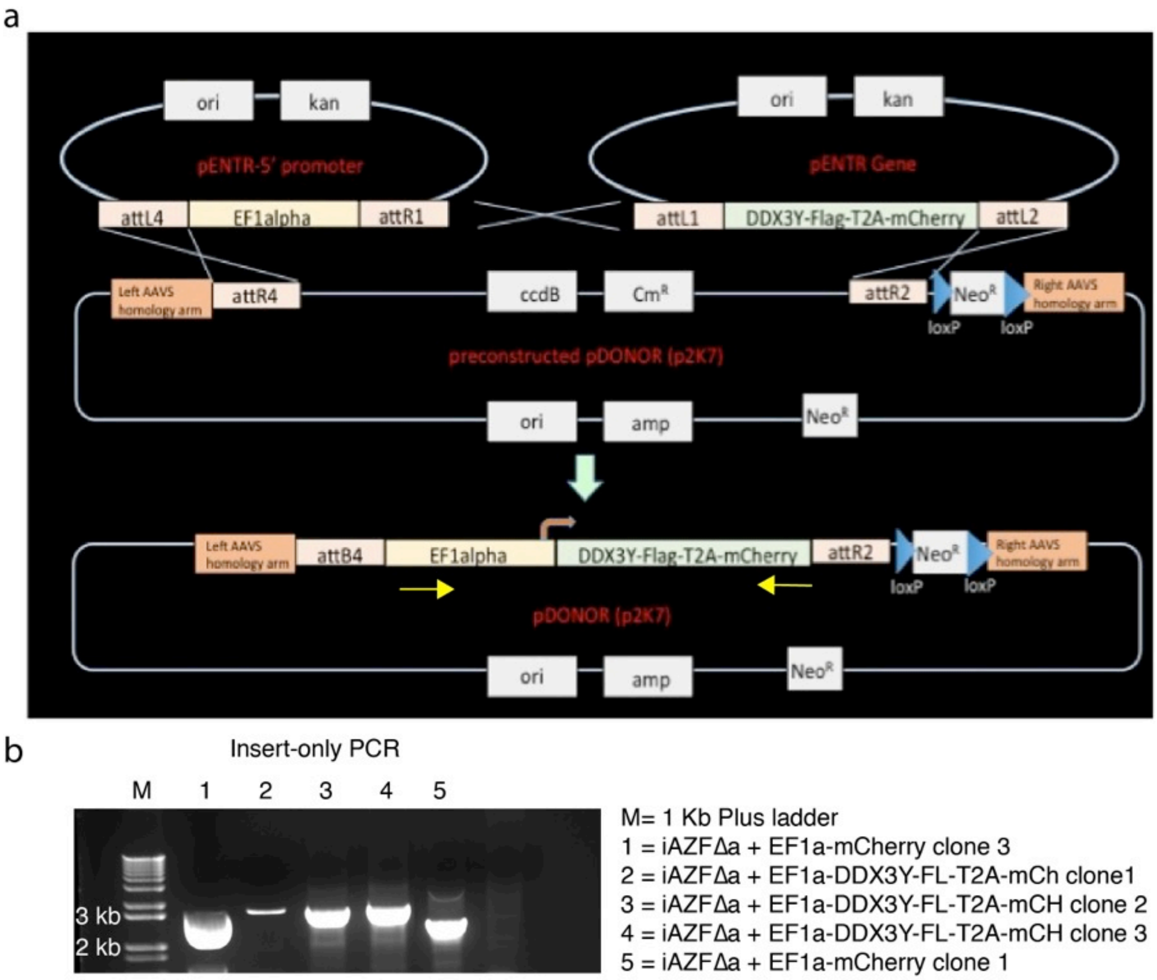

Supplementary Figure 1. Construction of DDX3Y targeting vector for homologous recombination.
a, A schematic diagram of the cloning scheme utilizing the Gateway® cloning technology to create a DDX3Y-FLAG-T2A-mCHERRY or mCHERRY alone under the control of a ubiquitous EF1α promoter.
b, Agarose gel image depicting correct insertion of the entire targeting vector into the genome of iAZFΔa iPSC clonal cell lines.

Supplementary Figure 2

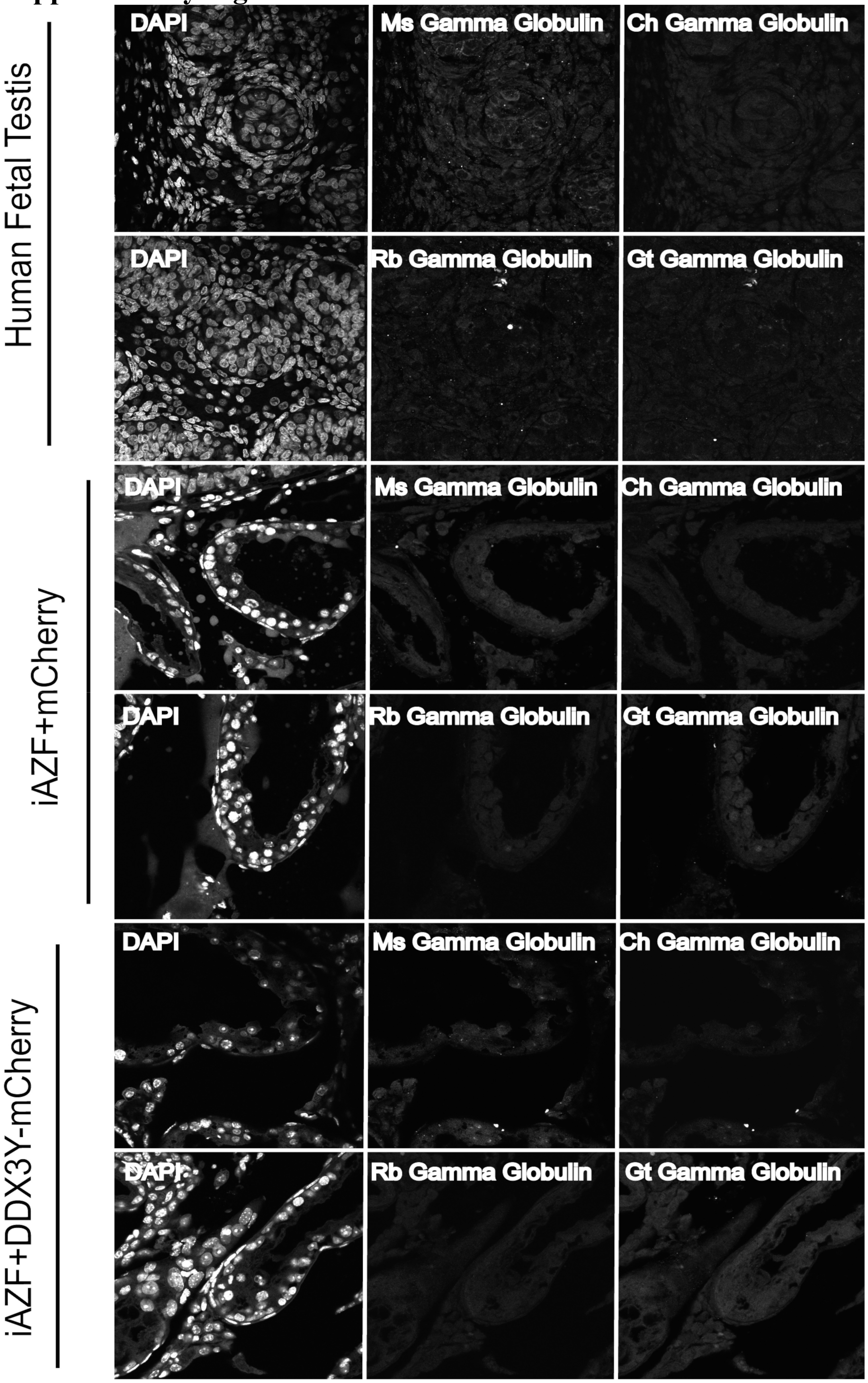

Supplementary Figure 2. Immunohistochemical analysis of isotype IgG antibodies. Isotype IgGs raised in Mouse, Chicken, Rabbit and Goat species were stained against sections from Human fetal testis, iAZFΔa+mCherry xenografted testes and iAZFΔa+DDX3Y-mCherry xenografted testis respectively. Panels depict grayscale images obtained for each IgG antibody. Nuclei corresponding to each region shown are counterstained with DAPI.

Supplementary Figure 3.

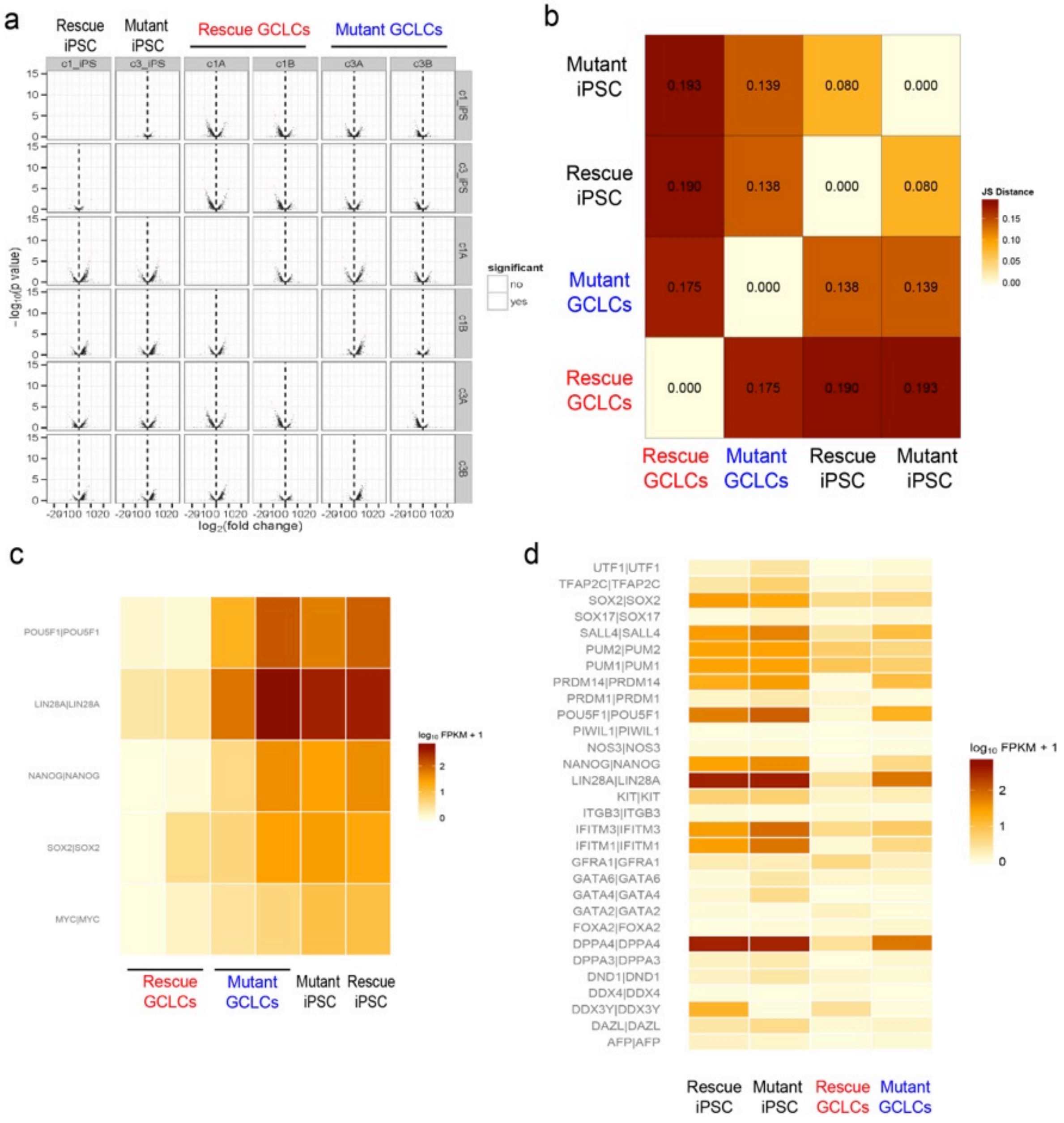

Supplementary Figure 3. Whole-genome RNA sequencing of donor iPSCs and xenograft-derived GCLCs.

a-b, Pairwise comparison between all donor iPSCs, two mutant cell line (iAZFΔa+mCHERRY) derived GCLC samples and two rescue cell line (iAZFΔa+DDX3Y-FL-mCHERRY) derived GCLC samples volcano plots and Jensen Shannon distances.

c, A heatmap of differential gene expression of pluripotency-associated genes in all samples.

d, A heatmap of differentiatial gene expression of Human primordial germ cell genes in all samples.

Supplementary Table 1. List of Genes and Transcripts Upregulated in Rescue GCLCs vs Mutant GCLCs (Cluster 1)

Gene Ensembl ID	Gene Symbol	Gene Name	PANTHER Family / Sub-family	Protein Class	Species
HUMAN|Ensembl=ENSG00000170606|UniProtKB=P34932	HSPA4	Heat shock 70 kDa protein 4;HSPA4;ortholog	HEAT SHOCK 70 KDA PROTEIN 4 (PTHR19375:SF136)	Hsp70 family chaperone	Homo sapiens
HUMAN|Ensembl=ENSG00000116288|UniProtKB=Q99497	PARK7	Protein DJ-1;PARK7;ortholog	PROTEIN DJ-1 (PTHR11019:SF4)	transcription factor;cysteine protease;RNA binding protein;cysteine protease	Homo sapiens
HUMAN|Ensembl=ENSG00000179059|UniProtKB=Q96MM3	ZFP42	Zinc finger protein 42 homolog;ZFP42;ortholog	ZINC FINGER PROTEIN 42 HOMOLOG (PTHR14003:SF8)	zinc finger transcription factor	Homo sapiens
HUMAN|Ensembl=ENSG00000154478|UniProtKB=Q8NDV2	GPR26	G-protein coupled receptor 26;GPR26;ortholog	G-PROTEIN COUPLED RECEPTOR 26 (PTHR24237:SF27)		Homo sapiens
HUMAN|Ensembl=ENSG00000171806|UniProtKB=O95568	METTL18	Histidine protein methyltransferase 1 homolog;METTL18;ortholog	HISTIDINE PROTEIN METHYLTRANSFERASE 1 HOMOLOG (PTHR21095:SF0)		Homo sapiens
HUMAN|Ensembl=ENSG00000175352|UniProtKB=Q9NQ35	NRIP3	Nuclear receptor-interacting protein 3;NRIP3;ortholog	NUCLEAR RECEPTOR-INTERACTING PROTEIN 3 (PTHR12917:SF2)	transcription cofactor;aspartic protease;aspartic protease	Homo sapiens
HUMAN|Ensembl=ENSG00000035141|UniProtKB=Q96C01	FAM136A	Protein FAM136A;FAM136A;ortholog	PROTEIN FAM136A (PTHR21096:SF0)		Homo sapiens
HUMAN|Ensembl=ENSG00000266562|UniProtKB=Q5VT52	RPRD2	Regulation of nuclear pre-mRNA domain-containing protein 2;RPRD2;ortholog	REGULATION OF NUCLEAR PRE-MRNA DOMAIN-CONTAINING PROTEIN 2 (PTHR12460:SF0)	kinase inhibitor	Homo sapiens
HUMAN|Ensembl=ENSG00000163125|UniProtKB=Q5VT52	RPRD2	Regulation of nuclear pre-mRNA domain-containing protein 2;RPRD2;ortholog	No Panther Hit		Homo sapiens
HUMAN|Ensembl=ENSG00000189067|UniProtKB=Q99732	LITAF	Lipopolysaccharide-induced tumor necrosis factor-alpha factor;LITAF;ortholog	LIPOPOLYSACCHARIDE-INDUCED TUMOR NECROSIS FACTOR-ALPHA FACTOR (PTHR23292:SF2)	transcription factor	Homo sapiens
HUMAN|Ensembl=ENSG00000163961|UniProtKB=Q8IYW5	RNF168	E3 ubiquitin-protein ligase RNF168;RNF168;ortholog	E3 UBIQUITIN-PROTEIN LIGASE RNF168 (PTHR23328:SF1)		Homo sapiens
HUMAN|Ensembl=ENSG00000124571|UniProtKB=Q9HAV4	XPO5	Exportin-5;XPO5;ortholog	EXPORTIN-5 (PTHR11223:SF3)	receptor	Homo sapiens
HUMAN|Ensembl=ENSG00000075340|UniProtKB=P35612	ADD2	Beta-adducin;ADD2;ortholog	BETA-ADDUCIN (PTHR10672:SF6)	non-motor actin binding protein	Homo sapiens
HUMAN|Ensembl=ENSG00000123545|UniProtKB=Q9P032	NDUFAF4	NADH dehydrogenase [ubiquinone] 1 alpha subcomplex assembly factor 4;NDUFAF4;ortholog	NADH DEHYDROGENASE [UBIQUINONE] 1 ALPHA SUBCOMPLEX ASSEMBLY FACTOR 4 (PTHR13338:SF4)		Homo sapiens
HUMAN|Ensembl=ENSG00000133835|UniProtKB=P51659	HSD17B4	Peroxisomal multifunctional enzyme type 2;HSD17B4;ortholog	PEROXISOMAL MULTIFUNCTIONAL ENZYME TYPE 2 (PTHR24316:SF2)	dehydrogenase;reductase	Homo sapiens
HUMAN|Ensembl=ENSG00000048028|UniProtKB=Q96RU2	USP28	Ubiquitin carboxyl-terminal hydrolase 28;USP28;ortholog	UBIQUITIN CARBOXYL-TERMINAL HYDROLASE 28 (PTHR24006:SF110)	cysteine protease;mRNA splicing factor;ubiquitin-protein ligase;cysteine protease	Homo sapiens
HUMAN|Ensembl=ENSG00000083817|UniProtKB=Q9BWM5	ZNF416	Zinc finger protein 416;ZNF416;ortholog	ZINC FINGER PROTEIN 416 (PTHR24402:SF15)	KRAB box transcription factor	Homo sapiens
HUMAN|Ensembl=ENSG00000136819|UniProtKB=Q9NZ63	C9orf78	Uncharacterized protein C9orf78;C9orf78;ortholog	NOVEL PROTEIN (ZGC:103692) (PTHR13486:SF2)		Homo sapiens
HUMAN|Ensembl=ENSG00000110906|UniProtKB=Q9H3F6	KCTD10	BTB/POZ domain-containing adapter for CUL3-mediated RhoA degradation protein 3;KCTD10;ortholog	BTB/POZ DOMAIN-CONTAINING ADAPTER FOR CUL3-MEDIATED RHOA DEGRADATION PROTEIN 3 (PTHR11145:SF14)		Homo sapiens
HUMAN|Ensembl=ENSG00000155640|UniProtKB=Q8N655	C10orf12	Uncharacterized protein C10orf12;C10orf12;ortholog	PROTEIN GM340 (PTHR14931:SF2)		Homo sapiens
HUMAN|Ensembl=ENSG00000173660|UniProtKB=P07919	UQCRH	Cytochrome b-c1 complex subunit 6, mitochondrial;UQCRH;ortholog	CYTOCHROME B-C1 COMPLEX SUBUNIT 6, MITOCHONDRIAL (PTHR15336:SF0)	reductase	Homo sapiens
HUMAN|Ensembl=ENSG00000147596|UniProtKB=Q9GZV8	PRDM14	PR domain zinc finger protein 14;PRDM14;ortholog	PR DOMAIN ZINC FINGER PROTEIN 14 (PTHR11389:SF354)	zinc finger transcription factor;DNA binding protein	Homo sapiens
HUMAN|Ensembl=ENSG00000131773|UniProtKB=O75525	KHDRBS3	KH domain-containing, RNA-binding, signal transduction-associated protein 3;KHDRBS3;ortholog	KH DOMAIN-CONTAINING, RNA-BINDING, SIGNAL TRANSDUCTION-ASSOCIATED PROTEIN 3 (PTHR11208:SF29)	transcription cofactor;mRNA splicing factor	Homo sapiens
HUMAN|Ensembl=ENSG00000179331|UniProtKB=Q14964	RAB39A	Ras-related protein Rab-39A;RAB39A;ortholog	RAS-RELATED PROTEIN RAB-39A (PTHR24073:SF312)		Homo sapiens
HUMAN|Ensembl=ENSG00000152439|UniProtKB=Q6PK81	ZNF773	Zinc finger protein 773;ZNF773;ortholog	ZINC FINGER PROTEIN 773 (PTHR24387:SF40)	KRAB box transcription factor	Homo sapiens
HUMAN|Ensembl=ENSG00000117016|UniProtKB=Q9UJD0	RIMS3	Regulating synaptic membrane exocytosis protein 3;RIMS3;ortholog	REGULATING SYNAPTIC MEMBRANE EXOCYTOSIS PROTEIN 3 (PTHR12157:SF18)	G-protein modulator	Homo sapiens
HUMAN|Ensembl=ENSG00000184697|UniProtKB=P56747	CLDN6	Claudin-6;CLDN6;ortholog	CLAUDIN-6 (PTHR12002:SF41)	tight junction	Homo sapiens
HUMAN|Ensembl=ENSG00000099995|UniProtKB=Q15459	SF3A1	Splicing factor 3A subunit 1;SF3A1;ortholog	SPLICING FACTOR 3A SUBUNIT 1 (PTHR15316:SF1)	mRNA splicing factor	Homo sapiens
HUMAN|Ensembl=ENSG00000205213|UniProtKB=Q9BXB1	LGR4	Leucine-rich repeat-containing G-protein coupled receptor 4;LGR4;ortholog	LEUCINE-RICH REPEAT-CONTAINING G-PROTEIN COUPLED RECEPTOR 4 (PTHR24367:SF246)	receptor;extracellular matrix protein	Homo sapiens
HUMAN|Ensembl=ENSG00000160877|UniProtKB=Q96RE7	NACC1	Nucleus accumbens-associated protein 1;NACC1;ortholog	NUCLEUS ACCUMBENS-ASSOCIATED PROTEIN 1 (PTHR23228:SF56)	zinc finger transcription factor	Homo sapiens
HUMAN|Ensembl=ENSG00000103429|UniProtKB=Q9NZS9	BFAR	Bifunctional apoptosis regulator;BFAR;ortholog	BIFUNCTIONAL APOPTOSIS REGULATOR (PTHR15898:SF1)		Homo sapiens
HUMAN|Ensembl=ENSG00000102893|UniProtKB=Q93100	PHKB	Phosphorylase b kinase regulatory subunit beta;PHKB;ortholog	PHOSPHORYLASE B KINASE REGULATORY SUBUNIT BETA (PTHR10749:SF3)	kinase activator	Homo sapiens
HUMAN|Ensembl=ENSG00000102096|UniProtKB=Q9P1W9	PIM2	Serine/threonine-protein kinase pim-2;PIM2;ortholog	SERINE/THREONINE-PROTEIN KINASE PIM-2 (PTHR22984:SF2)	serine/threonine protein kinase receptor;serine/threonine protein kinase receptor;protein kinase	Homo sapiens
HUMAN|Ensembl=ENSG00000088325|UniProtKB=Q9ULW0	TPX2	Targeting protein for Xklp2;TPX2;ortholog	TARGETING PROTEIN FOR XKLP2 (PTHR14326:SF9)	non-motor microtubule binding protein	Homo sapiens
HUMAN|Ensembl=ENSG00000118965|UniProtKB=Q9P2L0	WDR35	WD repeat-containing protein 35;WDR35;ortholog	WD REPEAT-CONTAINING PROTEIN 35 (PTHR16517:SF1)		Homo sapiens
HUMAN|Ensembl=ENSG00000156049|UniProtKB=O95837	GNA14	Guanine nucleotide-binding protein subunit alpha-14;GNA14;ortholog	GUANINE NUCLEOTIDE-BINDING PROTEIN SUBUNIT ALPHA-14 (PTHR10218:SF57)	heterotrimeric G-protein	Homo sapiens
HUMAN|Ensembl=ENSG00000175066|UniProtKB=Q6ZS86	GK5	Putative glycerol kinase 5;GK5;ortholog	GLYCEROL KINASE 5-RELATED (PTHR10196:SF54)	carbohydrate kinase;carbohydrate kinase	Homo sapiens
HUMAN|Ensembl=ENSG00000100353|UniProtKB=O15371	EIF3D	Eukaryotic translation initiation factor 3 subunit D;EIF3D;ortholog	EUKARYOTIC TRANSLATION INITIATION FACTOR 3 SUBUNIT D (PTHR12399:SF0)	translation initiation factor	Homo sapiens
HUMAN|Ensembl=ENSG00000127688|UniProtKB=Q9H2C0	GAN	Gigaxonin;GAN;ortholog	GIGAXONIN (PTHR24412:SF167)		Homo sapiens
HUMAN|Ensembl=ENSG00000165732|UniProtKB=Q9NR30	DDX21	Nucleolar RNA helicase 2;DDX21;ortholog	NUCLEOLAR RNA HELICASE 2 (PTHR24031:SF197)	RNA helicase;translation initiation factor;helicase	Homo sapiens
HUMAN|Ensembl=ENSG00000179361|UniProtKB=Q8IVW6	ARID3B	AT-rich interactive domain-containing protein 3B;ARID3B;ortholog	AT-RICH INTERACTIVE DOMAIN-CONTAINING PROTEIN 3B (PTHR15348:SF3)	transcription factor;nucleic acid binding	Homo sapiens
HUMAN|Ensembl=ENSG00000141076|UniProtKB=Q969X6	CIRH1A	Cirhin;CIRH1A;ortholog	CIRHIN (PTHR22841:SF4)		Homo sapiens
HUMAN|Ensembl=ENSG00000258405|UniProtKB=Q96N58	ZNF578	Zinc finger protein 578;ZNF578;ortholog	ZINC FINGER PROTEIN 578-RELATED (PTHR24407:SF12)		Homo sapiens
HUMAN|Ensembl=ENSG00000121644|UniProtKB=Q9BSY9	DESI2	Desumoylating isopeptidase 2;DESI2;ortholog	DESUMOYLATING ISOPEPTIDASE 2 (PTHR12378:SF6)		Homo sapiens
HUMAN|Ensembl=ENSG00000143970|UniProtKB=Q76L83	ASXL2	Putative Polycomb group protein ASXL2;ASXL2;ortholog	POLYCOMB GROUP PROTEIN ASXL2-RELATED (PTHR13578:SF11)		Homo sapiens
HUMAN|Ensembl=ENSG00000165733|UniProtKB=Q14692	BMS1	Ribosome biogenesis protein BMS1 homolog;BMS1;ortholog	RIBOSOME BIOGENESIS PROTEIN BMS1 HOMOLOG (PTHR12858:SF2)		Homo sapiens
HUMAN|Ensembl=ENSG00000119862|UniProtKB=Q3ZCW2	LGALSL	Galectin-related protein;LGALSL;ortholog	GALECTIN-RELATED PROTEIN (PTHR11346:SF98)	signaling molecule;cell adhesion molecule	Homo sapiens
HUMAN|Ensembl=ENSG00000101407|UniProtKB=O43156	TTI1	TELO2-interacting protein 1 homolog;TTI1;ortholog	TELO2-INTERACTING PROTEIN 1 HOMOLOG (PTHR18460:SF3)		Homo sapiens
HUMAN|Ensembl=ENSG00000262261|UniProtKB=Q6P1K8	GTF2H2C	General transcription factor IIH subunit 2-like protein;GTF2H2C;ortholog	No Panther Hit		Homo sapiens
HUMAN|Ensembl=ENSG00000131914|UniProtKB=Q9H9Z2	LIN28A	Protein lin-28 homolog A;LIN28A;ortholog	PROTEIN LIN-28 HOMOLOG A (PTHR11544:SF31)	DNA binding protein	Homo sapiens
HUMAN|Ensembl=ENSG00000263977|UniProtKB=Q15427	SF3B4	Splicing factor 3B subunit 4;SF3B4;ortholog	SPLICING FACTOR 3B SUBUNIT 4 (PTHR24011:SF0)	transcription factor;DNA binding protein;mRNA polyadenylation factor;mRNA splicing factor;ribonucleoprotein	Homo sapiens
HUMAN|Ensembl=ENSG00000181038|UniProtKB=Q86XA0	METTL23	Methyltransferase-like protein 23;METTL23;ortholog	METHYLTRANSFERASE-LIKE PROTEIN 23 (PTHR14614:SF2)		Homo sapiens
HUMAN|Ensembl=ENSG00000101695|UniProtKB=Q96EQ8	RNF125	E3 ubiquitin-protein ligase RNF125;RNF125;ortholog	E3 UBIQUITIN-PROTEIN LIGASE RNF125 (PTHR13982:SF2)	ubiquitin-protein ligase	Homo sapiens
HUMAN|Ensembl=ENSG00000197445|UniProtKB=Q6ZP98	C16orf47	Putative uncharacterized protein C16orf47;C16orf47;ortholog			Homo sapiens
HUMAN|Ensembl=ENSG00000133980|UniProtKB=Q9H8Y1	VRTN	Vertnin;VRTN;ortholog	VERTNIN (PTHR16081:SF0)		Homo sapiens
HUMAN|Ensembl=ENSG00000004961|UniProtKB=P53701	HCCS	Cytochrome c-type heme lyase;HCCS;ortholog	CYTOCHROME C-TYPE HEME LYASE (PTHR12743:SF0)	lyase	Homo sapiens

HUMAN|Ensembl=ENSG00000117450|UniProtKB=Q06830	PRDX1	Peroxiredoxin-1;PRDX1;ortholog	PEROXIREDOXIN-1 (PTHR10681:SF75)	peroxidase	Homo sapiens
HUMAN|Ensembl=ENSG00000168209|UniProtKB=Q9NX09	DDIT4	DNA damage-inducible transcript 4 protein;DDIT4;ortholog	DNA DAMAGE-INDUCIBLE TRANSCRIPT 4 PROTEIN (PTHR12478:SF7)		Homo sapiens
HUMAN|Ensembl=ENSG00000143368|UniProtKB=Q15427	SF3B4	Splicing factor 3B subunit 4;SF3B4;ortholog	No Panther Hit		Homo sapiens
HUMAN|Ensembl=ENSG00000176915|UniProtKB=Q86XL3	ANKLE2	Ankyrin repeat and LEM domain-containing protein 2;ANKLE2;ortholog	ANKYRIN REPEAT AND LEM DOMAIN-CONTAINING PROTEIN 2 (PTHR20976:SF1)		Homo sapiens
HUMAN|Ensembl=ENSG00000133393|UniProtKB=Q96NB1	FOPNL	LisH domain-containing protein FOPNL;FOPNL;ortholog	LISH DOMAIN-CONTAINING PROTEIN FOPNL (PTHR15431:SF4)		Homo sapiens
HUMAN|Ensembl=ENSG00000126870|UniProtKB=Q8WVS4	WDR60	WD repeat-containing protein 60;WDR60;ortholog	WD REPEAT-CONTAINING PROTEIN 60 (PTHR12442:SF30)	microtubule family cytoskeletal protein	Homo sapiens
HUMAN|Ensembl=ENSG00000121570|UniProtKB=Q7L190	DPPA4	Developmental pluripotency-associated protein 4;DPPA4;ortholog	DEVELOPMENTAL PLURIPOTENCY-ASSOCIATED PROTEIN 4 (PTHR16073:SF8)		Homo sapiens
HUMAN|Ensembl=ENSG00000091409|UniProtKB=P23229	ITGA6	Integrin alpha-6;ITGA6;ortholog	INTEGRIN ALPHA-6 (PTHR23220:SF9)	cell adhesion molecule	Homo sapiens
HUMAN|Ensembl=ENSG00000172318|UniProtKB=Q9Y5Z6	B3GALT1	Beta-1,3-galactosyltransferase 1;B3GALT1;ortholog	BETA-1,3-GALACTOSYLTRANSFERASE 1 (PTHR11214:SF20)	glycosyltransferase	Homo sapiens
HUMAN|Ensembl=ENSG00000100865|UniProtKB=Q9BW66	CINP	Cyclin-dependent kinase 2-interacting protein;CINP;ortholog	CYCLIN-DEPENDENT KINASE 2-INTERACTING PROTEIN (PTHR15827:SF2)		Homo sapiens
HUMAN|Ensembl=ENSG00000255823|UniProtKB=P0CJ75	MTRNR2L8	Humanin-like protein 8;MTRNR2L8;ortholog			Homo sapiens
HUMAN|Ensembl=ENSG00000155846|UniProtKB=Q86YN6	PPARGC1B	Peroxisome proliferator-activated receptor gamma coactivator 1-beta;PPARGC1B;ortholog	PEROXISOME PROLIFERATOR-ACTIVATED RECEPTOR GAMMA COACTIVATOR 1-BETA (PTHR15528:SF12)	transcription cofactor	Homo sapiens
HUMAN|Ensembl=ENSG00000183474|UniProtKB=Q6P1K8	GTF2H2C	General transcription factor IIH subunit 2-like protein;GTF2H2C;ortholog	GENERAL TRANSCRIPTION FACTOR IIH SUBUNIT 2-RELATED (PTHR12695:SF2)	transcription factor	Homo sapiens
HUMAN|Ensembl=ENSG00000013810|UniProtKB=Q9Y6A5	TACC3	Transforming acidic coiled-coil-containing protein 3;TACC3;ortholog	TRANSFORMING ACIDIC COILED-COIL-CONTAINING PROTEIN 3 (PTHR13924:SF4)		Homo sapiens
HUMAN|Ensembl=ENSG00000204514|UniProtKB=B7Z6K7	ZNF814	Putative uncharacterized zinc finger protein 814;ZNF814;ortholog	SUBFAMILY NOT NAMED (PTHR24377:SF3)	KRAB box transcription factor	Homo sapiens
HUMAN|Ensembl=ENSG00000164265|UniProtKB=Q96PL1	SCGB3A2	Secretoglobin family 3A member 2;SCGB3A2;ortholog			Homo sapiens
HUMAN|Ensembl=ENSG00000111704|UniProtKB=Q9H9S0	NANOG	Homeobox protein NANOG;NANOG;ortholog	HOMEOBOX PROTEIN NANOG-RELATED (PTHR24327:SF29)	homeobox transcription factor;DNA binding protein	Homo sapiens
HUMAN|Ensembl=ENSG00000175792|UniProtKB=Q9Y265	RUVBL1	RuvB-like 1;RUVBL1;ortholog	RUVB-LIKE 1 (PTHR11093:SF6)		Homo sapiens
HUMAN|Ensembl=ENSG00000105649|UniProtKB=P20336	RAB3A	Ras-related protein Rab-3A;RAB3A;ortholog	RAS-RELATED PROTEIN RAB-3A (PTHR24073:SF433)		Homo sapiens

Supplementary Table 2. List of Genes and Transcripts Downregulated in Rescue GCLCs vs Mutant GCLCs (Cluster 2)

Gene Ensembl ID	Gene Symbol	Gene Name	PANTHER Family / Sub-family	Protein Class	Species
HUMAN|Ensembl=ENSG00000164124|UniProtKB=Q7Z5S9	TMEM144	Transmembrane protein 144;TMEM144;ortholog	TRANSMEMBRANE PROTEIN 144 (PTHR16119:SF12)		Homo sapiens
HUMAN|Ensembl=ENSG00000140798|UniProtKB=Q96J65	ABCC12	Multidrug resistance-associated protein 9;ABCC12;ortholog	MULTIDRUG RESISTANCE-ASSOCIATED PROTEIN 9 (PTHR24223:SF10)	ATP-binding cassette (ABC) transporter	Homo sapiens
HUMAN|Ensembl=ENSG00000109132|UniProtKB=Q99453	PHOX2B	Paired mesoderm homeobox protein 2B;PHOX2B;ortholog	PAIRED MESODERM HOMEOBOX PROTEIN 2B (PTHR24329:SF301)	homeobox transcription factor;DNA binding protein	Homo sapiens
HUMAN|Ensembl=ENSG00000133110|UniProtKB=Q15063	POSTN	Periostin;POSTN;ortholog	PERIOSTIN (PTHR10900:SF12)	signaling molecule;cell adhesion molecule	Homo sapiens
HUMAN|Ensembl=ENSG00000171560|UniProtKB=P02671	FGA	Fibrinogen alpha chain;FGA;ortholog	FIBRINOGEN ALPHA CHAIN (PTHR19143:SF172)	signaling molecule	Homo sapiens
HUMAN|Ensembl=ENSG00000120129|UniProtKB=P28562	DUSP1	Dual specificity protein phosphatase 1;DUSP1;ortholog	DUAL SPECIFICITY PROTEIN PHOSPHATASE 1 (PTHR10159:SF309)	protein phosphatase;protein phosphatase;kinase inhibitor	Homo sapiens
HUMAN|Ensembl=ENSG00000101265|UniProtKB=P50749	RASSF2	Ras association domain-containing protein 2;RASSF2;ortholog	RAS ASSOCIATION DOMAIN-CONTAINING PROTEIN 2 (PTHR22738:SF14)	G-protein modulator	Homo sapiens
HUMAN|Ensembl=ENSG00000138772|UniProtKB=P12429	ANXA3	Annexin A3;ANXA3;ortholog	ANNEXIN A3 (PTHR10502:SF25)		Homo sapiens
HUMAN|Ensembl=ENSG00000169994|UniProtKB=Q6PIF6	MYO7B	Unconventional myosin-VIIb;MYO7B;ortholog	UNCONVENTIONAL MYOSIN-VIIB (PTHR13140:SF352)	G-protein modulator;actin binding motor protein;cell junction protein	Homo sapiens
HUMAN|Ensembl=ENSG00000187134|UniProtKB=Q04828	AKR1C1	Aldo-keto reductase family 1 member C1;AKR1C1;ortholog	ALDO-KETO REDUCTASE FAMILY 1 MEMBER C1 (PTHR11732:SF201)	reductase	Homo sapiens
HUMAN|Ensembl=ENSG00000081051|UniProtKB=P02771	AFP	Alpha-fetoprotein;AFP;ortholog	ALPHA-FETOPROTEIN (PTHR11385:SF7)	transfer/carrier protein	Homo sapiens
HUMAN|Ensembl=ENSG00000114113|UniProtKB=P50120	RBP2	Retinol-binding protein 2;RBP2;ortholog	RETINOL-BINDING PROTEIN 2 (PTHR11955:SF59)	transfer/carrier protein	Homo sapiens
HUMAN|Ensembl=ENSG00000091513|UniProtKB=P02787	TF	Serotransferrin;TF;ortholog	SEROTRANSFERRIN (PTHR11485:SF23)	transfer/carrier protein	Homo sapiens
HUMAN|Ensembl=ENSG00000146197|UniProtKB=Q8IX30	SCUBE3	Signal peptide, CUB and EGF-like domain-containing protein 3;SCUBE3;ortholog	SIGNAL PEPTIDE, CUB AND EGF-LIKE DOMAIN-CONTAINING PROTEIN 3 (PTHR24046:SF2)	extracellular matrix glycoprotein	Homo sapiens
HUMAN|Ensembl=ENSG00000120693|UniProtKB=O15198	SMAD9	Mothers against decapentaplegic homolog 9;SMAD9;ortholog	MOTHERS AGAINST DECAPENTAPLEGIC HOMOLOG 9 (PTHR13703:SF37)	transcription factor	Homo sapiens
HUMAN|Ensembl=ENSG00000138829|UniProtKB=P35556	FBN2	Fibrillin-2;FBN2;ortholog	FIBRILLIN-2 (PTHR24039:SF19)	signaling molecule;extracellular matrix glycoprotein;extracellular matrix structural protein;cell adhesion molecule;annexin;calmodulin	Homo sapiens
HUMAN|Ensembl=ENSG00000185559|UniProtKB=P80370	DLK1	Protein delta homolog 1;DLK1;ortholog	PROTEIN DELTA HOMOLOG 1 (PTHR24052:SF10)	membrane-bound signaling molecule;receptor;extracellular matrix structural protein	Homo sapiens
HUMAN|Ensembl=ENSG00000266592|UniProtKB=Q04828	AKR1C1	Aldo-keto reductase family 1 member C1;AKR1C1;ortholog	No Panther Hit		Homo sapiens
HUMAN|Ensembl=ENSG00000197445|UniProtKB=Q6ZP98	C16orf47	Putative uncharacterized protein C16orf47;C16orf47;ortholog			Homo sapiens
HUMAN|Ensembl=ENSG00000117525|UniProtKB=P13726	TF	Tissue factor;F3;ortholog	TISSUE FACTOR (PTHR20859:SF22)	type I cytokine receptor;type II cytokine receptor;defense/immunity protein	Homo sapiens
HUMAN|Ensembl=ENSG00000009830|UniProtKB=Q9UKY4	POMT2	Protein O-mannosyl-transferase 2;POMT2;ortholog	PROTEIN O-MANNOSYL-TRANSFERASE 2 (PTHR10050:SF31)	glycosyltransferase	Homo sapiens
HUMAN|Ensembl=ENSG00000108821|UniProtKB=P02452	COL1A1	Collagen alpha-1(I) chain;COL1A1;ortholog	COLLAGEN ALPHA-1(I) CHAIN (PTHR24023:SF54)	transporter;surfactant;receptor;extracellular matrix structural protein;antibacterial response protein	Homo sapiens
HUMAN|Ensembl=ENSG00000189184|UniProtKB=Q9HCL0	PCDH18	Protocadherin-18;PCDH18;ortholog	PROTOCADHERIN-18 (PTHR24028:SF9)	cadherin	Homo sapiens
HUMAN|Ensembl=ENSG00000168542|UniProtKB=P02461	COL3A1	Collagen alpha-1(III) chain;COL3A1;ortholog	COLLAGEN ALPHA-1(III) CHAIN (PTHR24023:SF415)	transporter;surfactant;receptor;extracellular matrix structural protein;antibacterial response protein	Homo sapiens
HUMAN|Ensembl=ENSG00000118271|UniProtKB=P02766	TTR	Transthyretin;TTR;ortholog	TRANSTHYRETIN (PTHR10395:SF12)	transporter;transfer/carrier protein	Homo sapiens
HUMAN|Ensembl=ENSG00000153201|UniProtKB=P49792	RBP2	E3 SUMO-protein ligase RanBP2;RANBP2;ortholog	E3 SUMO-PROTEIN LIGASE RANBP2 (PTHR23138:SF8)	G-protein modulator	Homo sapiens

Supplementary Table 3. List of all Differentially Expressed Genes and Transcripts between Rescue GCLCs and Mutant GCLCs

Gene ID	Sample 1	Sample 2	Value Sample1	Value Sample2	Fold Change (Log2)	P Value	Significant
MTRNR2L8	Resc GCLC A	Mut GCLC A	0.254406	1225.98	12.2345	2.97E-08	yes
HSPA4	Resc GCLC A	Mut GCLC A	0.0623045	109.159	10.7748	0.000000178	yes
SCGB3A2	Resc GCLC B	Mut GCLC A	0.149243	434.495	11.5075	0.000000393	yes
ESRG	Resc GCLC A	Mut GCLC A	0.691228	383.763	9.11684	0.00000052	yes
TTI1	Resc GCLC A	Mut GCLC A	0.0630756	78.6559	10.2843	0.000000634	yes
CLDN6	Resc GCLC A	Mut GCLC A	0.285355	347.642	10.2506	0.000000689	yes
PRDM14	Resc GCLC B	Mut GCLC A	0.0681608	122.468	10.8112	0.000000733	yes
FAM136A	Resc GCLC A	Mut GCLC A	0.135464	152.533	10.137	0.000000924	yes
COL3A1	Resc GCLC A	Mut GCLC A	141.41	0.229962	-9.26427	0.00000101	yes
SMAD9	Resc GCLC A	Mut GCLC A	68.7267	0.0389117	-10.7865	0.00000197	yes
AFP	Resc GCLC A	Mut GCLC B	135.37	0.212894	-9.31256	0.00000203	yes
LOC730102	Resc GCLC B	Mut GCLC A	0.0178485	32.4143	10.8266	0.00000221	yes
DPPA4	Resc GCLC A	Mut GCLC A	1.11565	341.656	8.25852	0.00000243	yes
MTRNR2L8	Resc GCLC A	Mut GCLC B	0.254406	297.387	10.191	0.00000282	yes
GPR26	Resc GCLC B	Mut GCLC A	0.011193	13.5138	10.2376	0.00000309	yes
RUVBL1	Resc GCLC B	Mut GCLC A	0.963603	359.387	8.54288	0.00000338	yes
LGR4	Resc GCLC A	Mut GCLC A	0.0423305	29.9281	9.46559	0.00000508	yes
COL1A1	Resc GCLC A	Mut GCLC A	159.548	0.593597	-8.07029	0.00000607	yes
RBP2	Resc GCLC A	Mut GCLC B	156.471	0.114805	-10.4125	0.00000658	yes
RPRD2	Resc GCLC A	Mut GCLC A	0.0288149	18.9455	9.36082	0.0000066	yes
MAP3K14-AS1	Resc GCLC B	Mut GCLC A	0.625299	380.706	9.24991	0.00000664	yes
ARID3B	Resc GCLC A	Mut GCLC A	0.245502	90.6754	8.52883	0.00000804	yes
ANKLE2	Resc GCLC A	Mut GCLC A	0.12854	56.9561	8.79148	0.00000807	yes
ZFP42	Resc GCLC B	Mut GCLC A	0.0657946	49.4857	9.55483	0.00000809	yes
SCUBE3	Resc GCLC A	Mut GCLC A	33.6791	0.055233	-9.25211	0.00000946	yes
C9orf78	Resc GCLC A	Mut GCLC A	0.14989	86.8719	9.17885	0.0000104	yes
NACC1	Resc GCLC A	Mut GCLC A	0.109265	63.2182	9.17637	0.0000104	yes
CLDN6	Resc GCLC B	Mut GCLC A	1.15272	347.642	8.23642	0.0000113	yes
DPPA4	Resc GCLC B	Mut GCLC A	1.81341	341.656	7.55769	0.0000113	yes
POMT2	Resc GCLC A	Mut GCLC A	51.8905	0.128089	-8.66218	0.0000121	yes
HCCS	Resc GCLC A	Mut GCLC A	0.100187	88.5843	9.78821	0.0000125	yes
LITAF	Resc GCLC A	Mut GCLC A	0.208909	109.818	9.03803	0.0000138	yes
ZNF416	Resc GCLC B	Mut GCLC A	0.0734422	47.0737	9.3241	0.0000141	yes
LGALSL	Resc GCLC B	Mut GCLC A	0.204361	59.1948	8.17821	0.0000145	yes
EIF3D	Resc GCLC A	Mut GCLC A	0.163987	85.9326	9.03348	0.0000148	yes
GAN	Resc GCLC A	Mut GCLC A	0.0492497	25.6943	9.02712	0.0000151	yes
ZFP42	Resc GCLC A	Mut GCLC A	0.0950025	49.4857	9.02483	0.0000151	yes
KCTD10	Resc GCLC A	Mut GCLC A	0.0705593	36.2906	9.00654	0.0000157	yes
UQCRH	Resc GCLC A	Mut GCLC A	2.51115	738.154	8.19943	0.0000182	yes
ARGResc GCL(	Resc GCLC A	Mut GCLC A	0.025904	24.3708	9.87777	0.0000184	yes
TDGF1P3	Resc GCLC B	Mut GCLC A	0.348041	93.1412	8.06402	0.0000187	yes
NANOG	Resc GCLC B	Mut GCLC A	0.0565329	40.4402	9.48248	0.0000189	yes
TPX2	Resc GCLC A	Mut GCLC A	0.312534	73.586	7.87928	0.0000196	yes
PHOX2B	Resc GCLC A	Mut GCLC B	40.4451	0.0558039	-9.50138	0.0000201	yes
DDIT4	Resc GCLC A	Mut GCLC A	0.239785	114.776	8.90287	0.0000203	yes
HSD17B4	Resc GCLC A	Mut GCLC A	0.424586	107.269	7.98095	0.0000214	yes
LOC441666	Resc GCLC B	Mut GCLC A	0.064978	19.8454	8.25464	0.0000214	yes
ENPP1	Mut iPSC	Resc GCLC A	13.1677	0.0279536	-8.87976	0.0000215	yes
DESI2	Resc GCLC A	Mut GCLC A	0.155978	43.8112	8.13382	0.0000217	yes
TACC3	Resc GCLC A	Mut GCLC A	0.130034	60.8595	8.87045	0.0000221	yes
USP28	Resc GCLC A	Mut GCLC A	0.186924	46.0101	7.94336	0.0000228	yes
TDGF1P3	Resc GCLC A	Mut GCLC A	0.384698	93.1412	7.91955	0.0000228	yes
RNF168	Resc GCLC A	Mut GCLC A	0.0425716	19.6549	8.85078	0.000023	yes
DDX21	Resc GCLC A	Mut GCLC A	0.0856451	38.4899	8.81189	0.0000231	yes
SF3B4	Resc GCLC A	Mut GCLC A	0.198186	90.8369	8.84028	0.0000234	yes
METTL18	Resc GCLC B	Mut GCLC A	0.0316189	28.5689	9.81944	0.0000241	yes
PHKB	Resc GCLC A	Mut GCLC A	0.0760152	33.656	8.79036	0.0000242	yes
LINC00997	Resc GCLC A	Mut GCLC A	32.0701	0.0962139	-8.38077	0.0000243	yes
BFAR	Resc GCLC A	Mut GCLC A	0.0858396	38.7996	8.82018	0.0000247	yes

Supplementary Table 3 (cont)

ADD2	Resc GCLC A	Mut GCLC A	0.18814	82.0599	8.76873	0.0000247	yes
C16orf47	Resc GCLC B	Mut GCLC A	0.0567725	50.459	9.7957	0.0000251	yes
XPO5	Resc GCLC A	Mut GCLC A	0.0469172	20.858	8.79626	0.0000262	yes
FGA	Resc GCLC A	Mut GCLC B	69.1782	0.0669567	-10.0129	0.0000265	yes
POSTN	Resc GCLC A	Mut GCLC A	129.319	0.129105	-9.96816	0.0000268	yes
ANXA3	Resc GCLC A	Mut GCLC B	102.7	0.328051	-8.2903	0.0000269	yes
MYO7B	Resc GCLC A	Mut GCLC B	15.31	0.0203873	-9.55259	0.0000273	yes
OC10012934	Resc GCLC A	Resc GCLC B	24.1687	0.0280134	-9.75281	0.0000283	yes
ZNF773	Resc GCLC B	Mut GCLC A	0.107591	48.7773	8.82451	0.0000285	yes
DLK1	Resc GCLC A	Mut GCLC A	267.753	1.04348	-8.00335	0.0000322	yes
ARGResc GCL	Resc GCLC B	Mut GCLC A	0.0780724	24.3708	8.28613	0.0000323	yes
POMT2	Resc GCLC A	Mut GCLC B	51.8905	0.204412	-7.98784	0.0000324	yes
RAB39A	Resc GCLC B	Mut GCLC A	0.0579812	35.4861	9.25745	0.0000326	yes
ZNF578	Resc GCLC A	Mut GCLC A	0.0649935	19.4594	8.22595	0.0000327	yes
FOPNL	Resc GCLC A	Mut GCLC A	0.0952838	39.1948	8.68421	0.0000342	yes
SF3A1	Resc GCLC A	Mut GCLC A	0.0564008	46.8599	9.69842	0.000036	yes
PRDX1	Resc GCLC A	Mut GCLC A	0.572414	483.208	9.72137	0.0000363	yes
PIM2	Resc GCLC A	Mut GCLC A	0.161381	64.6394	8.6458	0.0000373	yes
WDR60	Resc GCLC A	Mut GCLC A	0.0686545	27.3552	8.63824	0.0000387	yes
NDUFAF4	Resc GCLC A	Mut GCLC A	0.0827213	32.0679	8.59866	0.0000422	yes
ABCC12	Resc GCLC A	Mut GCLC B	14.8004	0.0283489	-9.02813	0.0000422	yes
KHDRBS3	Resc GCLC B	Mut GCLC A	0.0339166	25.0009	9.52577	0.0000461	yes
RAB3A	Resc GCLC B	Mut GCLC A	0.386216	135.139	8.45082	0.0000468	yes
PARK7	Resc GCLC A	Mut GCLC A	0.389283	227.01	9.18772	0.0000475	yes
CINP	Resc GCLC B	Mut GCLC A	0.156379	84.3514	9.07523	0.0000482	yes
GK5	Resc GCLC A	Mut GCLC A	0.0205714	11.7751	9.16088	0.0000486	yes
RNF125	Resc GCLC A	Mut GCLC A	0.0376725	13.8219	8.51923	0.0000507	yes
GTF2H2C	Resc GCLC A	Mut GCLC A	0.0625673	42.4942	9.40764	0.0000512	yes
LIN28A	Resc GCLC A	Mut GCLC A	1.25546	776.923	9.27341	0.0000512	yes
DUSP1	Resc GCLC A	Mut GCLC A	102.505	0.380725	-8.07272	0.0000518	yes
NRIP3	Resc GCLC B	Mut GCLC A	0.0148571	10.538	9.47023	0.0000519	yes
PIM2	Resc GCLC B	Mut GCLC A	0.191754	64.6394	8.39702	0.0000531	yes
ITGA6	Resc GCLC A	Mut GCLC A	0.300921	45.2234	7.23154	0.0000548	yes
METTL23	Resc GCLC A	Mut GCLC A	0.529398	234.5	8.79102	0.0000554	yes
FBN2	Resc GCLC A	Mut GCLC A	14.7071	0.06832	-7.74999	0.0000603	yes
LOC285889	Resc GCLC A	Mut GCLC B	32.1033	0.0909271	-8.46379	0.0000606	yes
CIRH1A	Resc GCLC A	Mut GCLC A	0.124098	42.6326	8.42434	0.0000626	yes
GNA14	Resc GCLC B	Mut GCLC A	0.0503753	25.0823	8.95974	0.0000635	yes
TMEM144	Resc GCLC A	Mut GCLC B	16.1286	0.0352805	-8.83654	0.0000655	yes
VRTN	Resc GCLC B	Mut GCLC A	0.0274434	17.9251	9.3513	0.0000657	yes
WDR35	Resc GCLC A	Mut GCLC A	0.200896	29.8862	7.21689	0.0000677	yes
AKR1C1	Resc GCLC A	Mut GCLC B	35.9252	0.0607911	-9.20692	0.0000677	yes
C10orf12	Resc GCLC A	Mut GCLC A	0.0493095	16.4399	8.38112	0.0000699	yes
ESRG	Resc GCLC B	Mut GCLC A	1.09014	383.763	8.45956	0.0000706	yes
TTR	Resc GCLC A	Mut GCLC A	108.352	0.316903	-8.41747	0.0000711	yes
B3GALT1	Resc GCLC B	Mut GCLC A	0.0238024	15.2082	9.31953	0.0000717	yes
RIMS3	Resc GCLC B	Mut GCLC A	0.128046	25.4217	7.63325	0.0000737	yes
ASXL2	Resc GCLC A	Mut GCLC A	0.0941708	18.6427	7.62912	0.0000747	yes
TF	Resc GCLC A	Mut GCLC B	73.5411	0.409452	-7.48871	0.0000756	yes
C16orf47	Mut GCLC A	Mut GCLC B	50.459	0.105131	-8.90677	0.0000766	yes
BMS1	Resc GCLC A	Mut GCLC A	0.135314	21.6561	7.32232	0.0000776	yes
PCDH18	Resc GCLC A	Mut GCLC A	10.9872	0.0334534	-8.35946	0.0000791	yes
RASSF2	Resc GCLC A	Mut GCLC A	27.7208	0.0903868	-8.26064	0.0000795	yes
ZNF814	Resc GCLC A	Mut GCLC A	0.145529	24.9513	7.42166	0.000081	yes
AFP	Resc GCLC A	Resc GCLC B	135.37	0.0244283	-12.4361	3.73E-08	yes
MTRNR2L8	Mut iPSC	Resc GCLC A	1072.3	0.254406	-12.0413	4.97E-08	yes
MTRNR2L8	Resc iPSC	Resc GCLC A	900.212	0.254406	-11.7889	7.48E-08	yes
HSPA4	Mut iPSC	Resc GCLC A	132.504	0.0623045	-11.0544	8.59E-08	yes
HSPA4	Resc iPSC	Resc GCLC A	109.647	0.0623045	-10.7812	0.000000175	yes
COL3A1	Mut iPSC	Resc GCLC A	0.0491572	141.41	11.4902	0.000000232	yes
COL3A1	Resc iPSC	Resc GCLC A	0.0520408	141.41	11.408	0.000000238	yes
APELA	Resc iPSC	Resc GCLC A	140.49	0.155196	-9.82216	0.000000568	yes
TF	Resc iPSC	Resc GCLC A	0.0292521	73.5411	11.2958	0.00000061	yes
DLK1	Resc iPSC	Resc GCLC A	0.110408	267.753	11.2438	0.000000668	yes

Supplementary Table 3 (cont)

APELA	Resc iPSC	Resc GCLC B	140.49	0.190881	-9.52358	0.00000083	yes
MTRNR2L8	Resc GCLC A	Resc GCLC B	0.254406	357.431	10.4563	0.00000154	yes
DPPA4	Resc iPSC	Resc GCLC A	375.841	1.11565	-8.3961	0.00000186	yes
LIN28A	Mut iPSC	Resc GCLC A	392.633	1.25546	-8.28882	0.00000189	yes
LIN28A	Resc iPSC	Resc GCLC A	382.56	1.25546	-8.25133	0.00000212	yes
DPPA4	Mut iPSC	Resc GCLC A	351.867	1.11565	-8.30101	0.00000218	yes
VSNL1	Resc iPSC	Resc GCLC B	117.981	0.168018	-9.45573	0.00000293	yes
VSNL1	Mut iPSC	Resc GCLC B	117.232	0.168018	-9.44653	0.000003	yes
ZNF90	Mut iPSC	Resc GCLC A	52.5933	0.0976618	-9.07287	0.00000393	yes
PTPN14	Mut iPSC	Resc GCLC A	24.1318	0.0539947	-8.8039	0.00000399	yes
TF	Mut iPSC	Resc GCLC A	0.0807481	73.5411	9.83091	0.00000402	yes
AFP	Mut iPSC	Resc GCLC A	0.293488	135.37	8.8494	0.00000411	yes
ESRG	Mut iPSC	Resc GCLC A	209.226	0.691228	-8.24168	0.00000433	yes
EPPK1	Resc GCLC A	Resc GCLC B	31.6906	0.0293943	-10.0743	0.00000456	yes
IGF2	Resc iPSC	Resc GCLC A	0.343421	225.76	9.3606	0.00000481	yes
ESRG	Resc iPSC	Resc GCLC A	203.349	0.691228	-8.20058	0.00000482	yes
TDGF1P3	Mut iPSC	Resc GCLC B	133.026	0.348041	-8.57823	0.00000509	yes
SLC7A1	Mut iPSC	Resc GCLC A	41.453	0.0849367	-8.93087	0.00000576	yes
IGF2	Mut iPSC	Resc GCLC A	0.715909	225.76	8.3008	0.00000596	yes
TDGF1P3	Mut iPSC	Resc GCLC A	133.026	0.384698	-8.43376	0.0000062	yes
TDGF1	Mut iPSC	Resc GCLC B	546.08	0.503442	-10.0831	0.00000627	yes
PHOX2B	Resc GCLC A	Resc GCLC B	40.4451	0.0302045	-10.387	0.00000642	yes
LIN28A	Mut iPSC	Resc GCLC B	392.633	1.79316	-7.77453	0.00000666	yes
TDGF1P3	Resc iPSC	Resc GCLC B	121.439	0.348041	-8.44675	0.00000713	yes
LIN28A	Resc iPSC	Resc GCLC B	382.56	1.79316	-7.73704	0.00000743	yes
CLDN6	Resc iPSC	Resc GCLC A	182.155	0.285355	-9.31819	0.00000764	yes
RNF168	Resc iPSC	Resc GCLC A	26.5958	0.0425716	-9.28709	0.00000798	yes
TTR	Resc iPSC	Resc GCLC A	0.0898881	108.352	10.2353	0.00000838	yes
TDGF1P3	Resc iPSC	Resc GCLC A	121.439	0.384698	-8.30228	0.00000868	yes
RNF168	Mut iPSC	Resc GCLC A	25.9675	0.0425716	-9.2526	0.00000872	yes
DPPA4	Resc iPSC	Resc GCLC B	375.841	1.81341	-7.69527	0.00000878	yes
PTPN14	Resc iPSC	Resc GCLC A	19.3898	0.0539947	-8.48826	0.00000897	yes
TDGF1	Resc iPSC	Resc GCLC B	460.09	0.503442	-9.83588	0.00000929	yes
PSAT1	Mut iPSC	Resc GCLC A	177.093	0.217169	-9.67147	0.00000942	yes
ABCC12	Resc GCLC A	Resc GCLC B	14.8004	0.0123912	-10.2221	0.00000945	yes
RBP2	Mut iPSC	Resc GCLC A	0.132422	156.471	10.2065	0.00000957	yes
DLK1	Mut iPSC	Resc GCLC A	0.490358	267.753	9.09285	0.00000965	yes
POLR3G	Resc iPSC	Resc GCLC B	36.7979	0.0734015	-8.9696	0.00000983	yes
LINC00997	Resc GCLC A	Resc GCLC B	32.0701	0.0582015	-9.10596	0.00000983	yes
ZNF208	Resc iPSC	Mut iPSC	0.017916	10.6701	9.2181	0.00000992	yes
DPPA4	Mut iPSC	Resc GCLC B	351.867	1.81341	-7.60018	0.0000103	yes
VSNL1	Resc iPSC	Mut GCLC A	117.981	0.342548	-8.42804	0.0000112	yes
VSNL1	Mut iPSC	Mut GCLC A	117.232	0.342548	-8.41884	0.0000115	yes
CLDN6	Mut iPSC	Resc GCLC A	162.14	0.285355	-9.15026	0.0000116	yes
HHLA1	Mut iPSC	Mut GCLC B	28.252	0.0435834	-9.34036	0.0000117	yes
ABCC12	Resc iPSC	Resc GCLC A	0.0140257	14.8004	10.0434	0.0000128	yes
GPR26	Resc iPSC	Mut GCLC A	0.0245286	13.5138	9.10575	0.0000128	yes
VSNL1	Resc iPSC	Resc GCLC A	117.981	0.363526	-8.34228	0.0000129	yes
KDR	Mut iPSC	Resc GCLC A	45.7703	0.158606	-8.17282	0.0000129	yes
ENPP1	Resc iPSC	Resc GCLC A	15.1369	0.0279536	-9.08082	0.0000131	yes
VSNL1	Mut iPSC	Resc GCLC A	117.232	0.363526	-8.33309	0.0000132	yes
AFP	Resc iPSC	Resc GCLC A	0.501663	135.37	8.07598	0.0000133	yes
ZNF90	Resc iPSC	Resc GCLC A	36.8327	0.0976618	-8.55898	0.0000144	yes
COL15A1	Resc iPSC	Resc GCLC A	0.0157994	15.7551	9.96173	0.0000155	yes
MYO7B	Resc GCLC A	Resc GCLC B	15.31	0.0151572	-9.98026	0.0000162	yes
SLC7A1	Resc iPSC	Resc GCLC A	31.1281	0.0849367	-8.51762	0.0000164	yes
ZFP42	Mut iPSC	Resc GCLC B	40.6345	0.0657946	-9.27052	0.0000164	yes
VSIG10	Mut iPSC	Resc GCLC A	24.652	0.0487586	-8.98184	0.0000172	yes
NANOG	Mut iPSC	Resc GCLC B	41.9298	0.0565329	-9.53467	0.0000173	yes
LOC285889	Mut iPSC	Resc GCLC A	0.0438799	32.1033	9.51494	0.0000178	yes
LOC730102	Mut GCLC A	Mut GCLC B	32.4143	0.0580066	-9.1262	0.0000192	yes
VRTN	Mut iPSC	Resc GCLC B	26.8501	0.0274434	-9.93425	0.0000195	yes
ZNF773	Mut GCLC A	Mut GCLC B	48.7773	0.0892743	-9.09375	0.0000202	yes
POLR3G	Resc iPSC	Resc GCLC A	36.7979	0.108805	-8.40173	0.000021	yes

Supplementary Table 3 (cont)

FGA	Resc GCLC A	Resc GCLC B	69.1782	0.10682	-9.33899	0.0000211	yes
ZNF578	Resc GCLC B	Mut GCLC A	0.0524944	19.4594	8.53409	0.0000223	yes
HSD17B4	Mut iPSC	Resc GCLC A	105.859	0.424586	-7.96187	0.0000226	yes
GPR26	Mut iPSC	Mut GCLC A	0.0344261	13.5138	8.61671	0.0000233	yes
MUC6	Mut iPSC	Resc GCLC A	0.0891107	34.4286	8.59379	0.0000236	yes
SLC7A5	Mut iPSC	Resc GCLC A	74.9199	0.136415	-9.1012	0.0000236	yes
POLR3G	Mut iPSC	Resc GCLC B	28.4656	0.0734015	-8.59919	0.0000246	yes
APELA	Mut iPSC	Resc GCLC A	49.5301	0.155196	-8.31807	0.000026	yes
MYO7B	Resc iPSC	Resc GCLC A	0.0184647	15.31	9.69549	0.0000267	yes
NDUFA6	Resc GCLC A	Mut GCLC A	0.253985	111.813	8.78212	0.000027	yes
KAL1	Resc iPSC	Resc GCLC A	14.4113	0.032864	-8.77648	0.0000271	yes
MUC6	Resc iPSC	Resc GCLC A	0.0971408	34.4286	8.46932	0.0000285	yes
XPO5	Mut iPSC	Resc GCLC A	20.4676	0.0469172	-8.76901	0.000029	yes
OC10012934	Resc iPSC	Resc GCLC A	0.0289335	24.1687	9.70618	0.0000295	yes
FZD10	Resc iPSC	Resc GCLC A	0.0350574	28.6224	9.67321	0.0000299	yes
DHCR24	Mut iPSC	Resc GCLC A	45.7791	0.147064	-8.2821	0.0000299	yes
ZFP42	Mut iPSC	Resc GCLC A	40.6345	0.0950025	-8.74052	0.0000305	yes
AASS	Resc iPSC	Resc GCLC A	208.316	1.56931	-7.0525	0.0000356	yes
ANXA3	Resc GCLC A	Resc GCLC B	102.7	0.373604	-8.10271	0.0000358	yes
FAM129A	Resc iPSC	Resc GCLC B	6.12275	0.00762531	-9.64917	0.000036	yes
KDR	Mut iPSC	Resc GCLC B	45.7703	0.24975	-7.51779	0.000037	yes
ZFP42	Resc iPSC	Resc GCLC B	31.8892	0.0657946	-8.92088	0.0000372	yes
APELA	Mut iPSC	Resc GCLC B	49.5301	0.190881	-8.01949	0.0000379	yes
DDX21	Resc iPSC	Resc GCLC A	32.9247	0.0856451	-8.58658	0.0000389	yes
ZNF208	Mut iPSC	Resc GCLC A	10.6701	0.0376802	-8.14555	0.0000398	yes
PSAT1	Resc iPSC	Resc GCLC A	106.071	0.217169	-8.93199	0.0000449	yes
SERPINB9	Mut iPSC	Resc GCLC B	67.4353	0.404293	-7.38196	0.0000475	yes
USP44	Resc iPSC	Resc GCLC B	62.988	0.0341749	-10.8479	0.0000482	yes
TPX2	Mut iPSC	Resc GCLC A	57.1137	0.312534	-7.51368	0.0000493	yes
DCN	Mut iPSC	Resc GCLC A	0.0218381	76.1359	11.7675	0.0000494	yes
VSIG10	Resc iPSC	Resc GCLC A	18.0395	0.0487586	-8.53129	0.0000505	yes
POLR3G	Mut iPSC	Resc GCLC A	28.4656	0.108805	-8.03132	0.0000519	yes
SCD	Resc iPSC	Resc GCLC A	240.721	2.0088	-6.90488	0.0000526	yes
TTC9	Resc iPSC	Resc GCLC A	18.0672	0.0497233	-8.50523	0.0000532	yes
DESI2	Mut iPSC	Resc GCLC A	33.8406	0.155978	-7.76127	0.0000541	yes
H19	Mut iPSC	Resc GCLC A	0.772456	197.745	7.99997	0.0000551	yes
FAM136A	Mut iPSC	Resc GCLC A	48.3349	0.135464	-8.47901	0.0000564	yes
HADHA	Mut iPSC	Resc GCLC A	44.2588	0.171134	-8.0147	0.0000567	yes
HHLA1	Mut iPSC	Mut GCLC A	28.252	0.107393	-8.03931	0.0000573	yes
SCD	Mut iPSC	Resc GCLC A	234.044	2.0088	-6.8643	0.0000574	yes
DKK2	Resc iPSC	Resc GCLC A	0.0149476	10.0679	9.39564	0.0000582	yes
GSTA2	Mut iPSC	Resc GCLC A	0.0388233	26.5631	9.41829	0.0000587	yes
PROS1	Resc GCLC A	Resc GCLC B	9.19882	0.0134415	-9.41861	0.0000592	yes
VRTN	Resc iPSC	Resc GCLC B	19.0441	0.0274434	-9.43866	0.0000593	yes
USP28	Mut iPSC	Resc GCLC A	34.7598	0.186924	-7.53883	0.0000626	yes
SLC35B4	Mut iPSC	Resc GCLC A	16.5915	0.0668001	-7.95637	0.0000634	yes
DESI2	Resc iPSC	Resc GCLC A	32.2568	0.155978	-7.69212	0.0000637	yes
LGR4	Resc iPSC	Resc GCLC A	14.3515	0.0423305	-8.40529	0.000065	yes
APOB	Resc GCLC A	Resc GCLC B	10.6098	0.0579958	-7.51524	0.0000679	yes
ZFP42	Resc iPSC	Resc GCLC A	31.8892	0.0950025	-8.39089	0.0000689	yes
GINS1	Mut iPSC	Resc GCLC A	32.7478	0.136545	-7.90588	0.0000712	yes
HADHA	Resc iPSC	Resc GCLC A	41.4738	0.171134	-7.92093	0.0000716	yes
MPHOSPH10	Mut iPSC	Resc GCLC A	49.7394	0.247359	-7.65164	0.0000717	yes
FAM129A	Mut iPSC	Resc GCLC B	4.90587	0.00762531	-9.3295	0.0000721	yes
KAL1	Mut iPSC	Resc GCLC A	10.8034	0.032864	-8.36076	0.0000722	yes
ZNF90	Mut iPSC	Resc GCLC B	52.5933	0.376816	-7.12488	0.000074	yes
HSD17B12	Resc iPSC	Resc GCLC A	69.167	0.43237	-7.32167	0.0000773	yes
NANOG	Resc iPSC	Resc GCLC B	26.5436	0.0565329	-8.87506	0.0000773	yes
DHCR24	Resc iPSC	Resc GCLC A	34.8167	0.147064	-7.88719	0.0000785	yes
USP44	Resc iPSC	Resc GCLC A	62.988	0.150613	-8.70809	0.0000799	yes
TF	Resc GCLC A	Resc GCLC B	73.5411	0.411728	-7.48071	0.0000802	yes
